# A putative E3 ubiquitin ligase substrate receptor degrades transcription factor SmNAC to enhance bacterial wilt resistance in eggplant

**DOI:** 10.1093/hr/uhad246

**Published:** 2023-11-27

**Authors:** Shuangshuang Yan, Yixi Wang, Bingwei Yu, Yuwei Gan, Jianjun Lei, Changming Chen, Zhangsheng Zhu, Zhengkun Qiu, Bihao Cao

**Affiliations:** Key Laboratory of Biology and Genetic Improvement of Horticultural Crops, Ministry of Agriculture and Rural Affairs/Guangdong Vegetable Engineering and Technology Research Center, Guangdong Provincial Key Laboratory of Postharvest Science of Fruits and Vegetables, College of Horticulture, South China Agricultural University, Guangzhou 510642, China; Key Laboratory of Biology and Genetic Improvement of Horticultural Crops, Ministry of Agriculture and Rural Affairs/Guangdong Vegetable Engineering and Technology Research Center, Guangdong Provincial Key Laboratory of Postharvest Science of Fruits and Vegetables, College of Horticulture, South China Agricultural University, Guangzhou 510642, China; Key Laboratory of Biology and Genetic Improvement of Horticultural Crops, Ministry of Agriculture and Rural Affairs/Guangdong Vegetable Engineering and Technology Research Center, Guangdong Provincial Key Laboratory of Postharvest Science of Fruits and Vegetables, College of Horticulture, South China Agricultural University, Guangzhou 510642, China; Key Laboratory of Biology and Genetic Improvement of Horticultural Crops, Ministry of Agriculture and Rural Affairs/Guangdong Vegetable Engineering and Technology Research Center, Guangdong Provincial Key Laboratory of Postharvest Science of Fruits and Vegetables, College of Horticulture, South China Agricultural University, Guangzhou 510642, China; Key Laboratory of Biology and Genetic Improvement of Horticultural Crops, Ministry of Agriculture and Rural Affairs/Guangdong Vegetable Engineering and Technology Research Center, Guangdong Provincial Key Laboratory of Postharvest Science of Fruits and Vegetables, College of Horticulture, South China Agricultural University, Guangzhou 510642, China; Key Laboratory of Biology and Genetic Improvement of Horticultural Crops, Ministry of Agriculture and Rural Affairs/Guangdong Vegetable Engineering and Technology Research Center, Guangdong Provincial Key Laboratory of Postharvest Science of Fruits and Vegetables, College of Horticulture, South China Agricultural University, Guangzhou 510642, China; Key Laboratory of Biology and Genetic Improvement of Horticultural Crops, Ministry of Agriculture and Rural Affairs/Guangdong Vegetable Engineering and Technology Research Center, Guangdong Provincial Key Laboratory of Postharvest Science of Fruits and Vegetables, College of Horticulture, South China Agricultural University, Guangzhou 510642, China; Key Laboratory of Biology and Genetic Improvement of Horticultural Crops, Ministry of Agriculture and Rural Affairs/Guangdong Vegetable Engineering and Technology Research Center, Guangdong Provincial Key Laboratory of Postharvest Science of Fruits and Vegetables, College of Horticulture, South China Agricultural University, Guangzhou 510642, China; Key Laboratory of Biology and Genetic Improvement of Horticultural Crops, Ministry of Agriculture and Rural Affairs/Guangdong Vegetable Engineering and Technology Research Center, Guangdong Provincial Key Laboratory of Postharvest Science of Fruits and Vegetables, College of Horticulture, South China Agricultural University, Guangzhou 510642, China

## Abstract

Bacterial wilt caused by *Ralstonia solanacearum* is a severe soil-borne disease globally, limiting the production in Solanaceae plants. SmNAC negatively regulated eggplant resistance to Bacterial wilt (BW) though restraining salicylic acid (SA) biosynthesis. However, other mechanisms through which SmNAC regulates BW resistance remain unknown. Here, we identified an interaction factor, SmDDA1b, encoding a substrate receptor for E3 ubiquitin ligase, from the eggplant cDNA library using SmNAC as bait. *SmDDA1b* expression was promoted by *R. solanacearum* inoculation and exogenous SA treatment. The virus-induced gene silencing of the *SmDDA1b* suppressed the BW resistance of eggplants; *SmDDA1b* overexpression enhanced the BW resistance of tomato plants. SmDDA1b positively regulates BW resistance by inhibiting the spread of *R. solanacearum* within plants. The SA content and the SA biosynthesis gene *ICS1* and signaling pathway genes decreased in the *SmDDA1b*-silenced plants but increased in *SmDDA1b*-overexpression plants. Moreover, SmDDB1 protein showed interaction with SmCUL4 and SmDDA1b and protein degradation experiments indicated that SmDDA1b reduced SmNAC protein levels through proteasome degradation. Furthermore, SmNAC could directly bind the *SmDDA1b* promoter and repress its transcription. Thus, SmDDA1b is a novel regulator functioning in BW resistance of solanaceous crops via the SmNAC-mediated SA pathway. Those results also revealed a negative feedback loop between SmDDA1b and SmNAC controlling BW resistance.

## Introduction

As a soil-borne bacterial disease, bacterial wilt (BW) is triggered by members of the *Ralstonia solanacearum* species complex (RSSC) [1]. it infects about 200 host plant species of 50 families, especially the Solanaceae family [2]. Generally, *R. solanacearum* secretes extracellular polysaccharides and proteases and self-reproduction in the plant vascular bundle; consequently, the water transport is blocked, which leads to plant death [3]. During crop production, bacterial wilt is difficult to control because *R. solanacearum* spreads through irrigation water and infected plants materials. Therefore, to investigate the genes involved in BW resistance is crucial in crop breeding.

Several genes regulating BW resistance have been identified in various plants. The first BW resistance gene is *RRS1-R* in *Arabidopsis* plants, it interacts with the matching PopP2 effector secreted by *R. solanacearum,* resulting in BW resistance [4]. In *Arabidopsis thaliana* ecotype Wassilewskija, RRS1 and RPS4 was involved in resistance to BW resistance in cruciferous crops [5]. When the elongation factor-Tu (EF-Tu) receptor (EFR) is ectopically expressed in potato (*Solanum tuberosum*) and in tomato (*Solanum lycopersicum*), the transgenic plants indicate reduced BW resistance [6, 7]. The histone deacetylase (HDAC)-mediated histone acetylation also suppress BW resistance in tomatoes [8]. In tomatoes, the BW resistance is elevated due to overexpressed potato *StNACb4* [9]. In tobacco (*Nicotiana tabacum*), the transcription factor bHLH93 boosted BW resistance by interacting with the *R. solanacearum* effector Ripl [10].

Ubiquitination has vital functions in plant disease resistance. In eukaryotes, protein degradation is mainly regulated by the conserved ubiquitin/26S proteasome system (UPS). ubiquitin-activating enzyme (E1) activates ubiquitin, then the ubiquitin binds to a ubiquitin-conjugating enzyme (E2) through a thiol ester bond. The target proteins are recruited by ubiquitin ligase (E3), and then degraded by ubiquitin, which is transferred by ubiquitin ligase (E3) [11]. The E3 ligases comprise three major groups: homologs to E6-associated protein C-terminus (HECT), really interesting new gene (RING), and plant U-box (PUB) [12].

RING E3 ligases consist of mono- and multi-subunit E3 ligases, which is the largest group. The cullin-RING ligases (CRLs) belong to the multi-subunit RING E3s [11]. CRLs are composed of the scaffold protein cullin, RING-containing protein RING-BOX (RBX)-1, an adaptor, and a substrate receptor [13]. There are five cullin proteins, including CUL1, CUL2, CUL3a, CUL3b, and CUL4, in plants [11]. Thereinto, CRL4 is involved in regulating cell biology and responding to various abiotic and biotic stresses [11]. The substrate recognition proteins DET1-associated protein 1 (DDA1) and cullin 4-related factors (DCFA) are incorporated into CRL4 by the DNA Damage-binding1 (DDB1) adaptor protein [14]. As a subunit of the plant DDB1-DET1-DDA1 (DDD) complex, DDA1 negatively regulates photomorphogenesis though interacting with COP10 [15]. In addition, DDA1, also as part of the COP10-DET1-DDB1 (CDD) complex, recognizes ubiquitination targets which impart substrate specificity for CRL4 and DDA1 desensitizes abscisic acid (ABA) signaling by regulating ABA receptor stability [16]. However, how CRL4 affects plant development and resistance to stress remains unclear.

**Figure 1:**
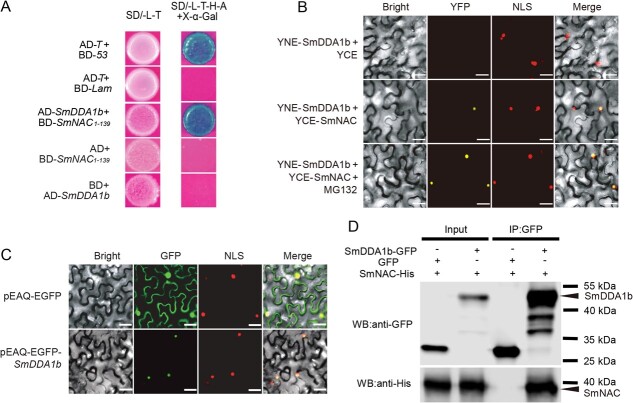
Interaction between SmDDA1b and SmNAC and the subcellular localization analysis of SmDDA1b. (**A**) Yeast two-hybrid (Y2H) assays of SmNAC and SmDDA1b. The co-transformed BD-53 and AD-T in the Y2H Gold strain were used as the positive control, while co-transformed BD-Lam and AD-T in the Y2H Gold strain were used as the negative control. SmNAC1–139 indicates the N-terminal 139 aa of SmNAC. (**B**) Bimolecular fluorescence complementation (BiFC) assays between SmDDA1b and SmNAC. YFP indicates the interaction between two proteins. NLS represents the nucleus location. (**C**) The subcellular localization analysis of SmDDA1b. GFP and NLS indicate the subcellular location of SmDDA1b in the nucleus. (**D**) Co-Immunoprecipitation (CoIP) analysis of SmDDA1b and SmNAC. Scale bar in (B-C) represents 50 μm.

Salicylic acid (SA) plays critical a part in local and systemic defense responsiveness to biotrophic pathogens, including *R. solanacearum* [17]. Isochorismate synthase (ICS1) is a predominantly catalyzing enzyme in the process of SA biosynthesis [18]. *EDS1*, *GluA*, *NPR1*, *TGA*, *SGT1*, and *PAD4*, which belongs to SA signaling pathway, participate in plant BW resistance [19–21]. For example, high-level SA triggers NPR1 deoligomerization and translocation to the nucleus. NPR1 induces the expression of *PR-1* by interaction with TGA and, consequently, systemic acquired resistance (SAR) is activated [17].

Although most solanaceous crops are susceptible to BW, several eggplant (*Solanum melongena*) cultivars have shown high levels of BW resistance, making them ideal crops for BW resistance analysis. Some BW resistance-related genes or loci, including *EBWR9* [22], *SmSPDS*, and *SmMYB44* [23] are identified in eggplants. SmNAC play a negative role in resistance to BW by repressing *SmICS1* expression in eggplants [24].

In this study, SmNAC protein was used as bait for screening the interactors in the eggplant cDNA library. The E3 ubiquitin ligase substrate receptor SmDDA1b was identified and found to positively regulate BW resistance and SA contents in eggplants. SmDDA1b also interacted with SmNAC to form a negative feedback loop (SmDDA1b-SmNAC) which regulated SA production, thus enhancing BW resistance in eggplant.

## Results

### SmDDA1b physically interacts with SmNAC

Our previous study demonstrated that *SmNAC* negatively regulates the BW resistance of eggplants by inhibiting SA biosynthesis [24]. As the bait protein, the 139-amino acid N-terminal portion of SmNAC (SmNAC_1–139_), containing a non-self-activating NAM domain, was used to screen the interaction factors in the cDNA library of eggplant leaves after BW inoculated. A putative E3 ubiquitin ligase substrate receptor, LOC102586503 (SmDDA1b hereafter), encoding 167 amino acids residues, was shown to interact with SmNAC (Fig. 1A). Based on its phylogeny and protein structure, we named it SmDDA1b. The interaction was confirmed by yeast two-hybrid (Y2H) assay (Fig. 1A). The bimolecular fluorescence complementation (BiFC) and CoIP assays also confirmed the interaction between SmDDA1b and SmNAC (Fig. 1B and D), implying that SmNAC indeed interacts with SmDDA1b.

SmDDA1b is a homolog of AtDDA1, a substrate receptor protein of CUL4-DDB1 type E3 ubiquitin ligase (CRL4) [16]. We retrieved DDA1 homologs from 15 representative dicotyledonous plants, and phylogenetic analysis showed that the DDA1 proteins clustered into two clades: the DDA1a lineage, which only had DDA1 domain in their protein, and the DDA1b lineage containing the DDA1 and SAP domains (Fig. S1A and B and Table S1, see online supplementary material). SmDDA1b and its homolog in solanaceous plants clustered into the DDA1b lineage (Fig. S1A, see online supplementary material). Moreover, green fluorescent protein (GFP)-tagged SmDDA1b was targeted to the nucleus (Fig. 1C). These results implied that SmDDA1b functions as a substrate receptor in eggplant.

### Transcriptional analysis of *SmDDA1b* in eggplant

**Figure 2:**
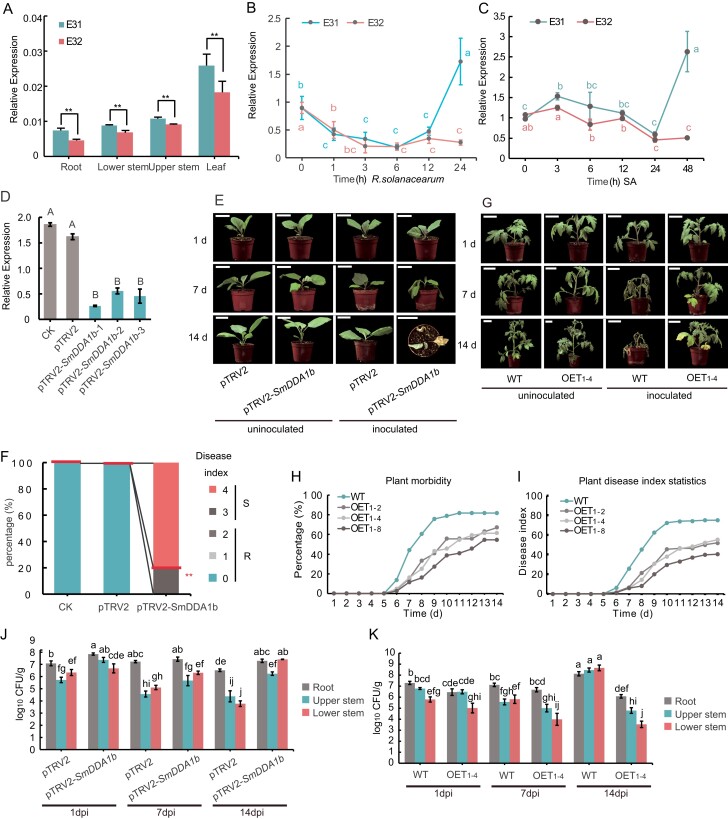
*SmDDA1b* expression and phenotypic analysis of *SmDDA1b-*silenced and *SmDDA1b*-overexpression plants inoculated with *Ralstonia solanacearum.* (**A**) The expression pattern of *SmDDA1b* in E31 and E32 tissues. Data are expressed as mean ± SD values (*n* = 3) (^*^P < 0.05; ^**^P < 0.01, according to the Student’s *t*-test). The reference gene was *SmActin*. (**B**) The expression pattern of *SmDDA1b* in E31 and E32 after inoculation with *R. solanacearum*. The reference gene was *SmActin*. (**C**) Relative expression of *SmDDA1b* in E31 and E32 after salicylic acid treatment. Data are expressed as mean ± SEM values of the three biological replicates. Different letters indicate statistically significant differences among the groups (Tukey’s honest significant difference test, *P* < 0.05). The reference gene was *SmActin*. (**D**) Relative expression of *SmDDA1b* in control plants and *SmDDA1b*-silenced plants. CK represents the group treated with water, while pTRV2 indicates the group treated with an empty vector solution. The pTRV2*-SmDDA1b* indicates the virus-induced gene silencing (VIGS)-treated plants. Each treatment had at least 10 biological replicates. Data are expressed as mean ± SEM values of the three biological replicates. Different letters indicate statistically significant differences among the groups (Tukey’s honest significant difference test, *P* < 0.01). The reference gene was *SmActin*. (**E**) The phenotypes of the control (pTRV2) and SmDDA1b silenced plants (pTRV2-*SmDDA1b*) at 1, 7, and 14 d post-inoculation with *R. solanacearum* in eggplants. Scale bars indicate 5 cm. (**F**) The disease index of control plants and *SmDDA1b*-silenced 10 d after inoculation with *R. solanacearum* in eggplants. The ordinate represents the percentage of the plants at each disease level. A total of ten eggplant seedlings were silenced. (**G**) The phenotypes of WT and *SmDDA1b*-overexpressing plants (OET1–4) at 1, 7, and 14 d post-inoculation with *R. solanacearum* in tomatoes. Scale bars indicate 5 cm. (**H**–**I**) The morbidity (**H**) and disease index (**I**) of WT and OE-*SmDDA1b* seedlings after infected with *R. solanacearum* at over 14 d in tomatoes. (**J**–**K**) The *R. solanacearum* colonization of control plants (pTRV2), *SmDDA1b*-silenced plants (pTRV2-*SmDDA1b*) (**J**) in eggplant, WT, and *SmDDA1b*-overexpressing plants in tomatoes (OET1–4) (**K**). The samples (root, lower stem, and upper stem) were obtained at 1, 7, and 14 d post-inoculation with *R. solanacearum*. Data are expressed as mean ± SEM values of the three biological replicates. Different letters indicate statistically significant differences among the groups (Tukey’s honest significant difference test, *P* < 0.05).

Because SmNAC regulates BW resistance in eggplants via the SA pathway [24], we evaluated whether SmDDA1b could also be involved in resistance to BW. No differential nucleotide sites were found between the *SmDDA1b* cDNA and genomic DNA sequences of BW-resistant line E31 (R) and BW-susceptible line E32 (S) of eggplants (Fig. S2A and C, see online supplementary material). While 270 bp, which contained three NAC binding *cis*-acting elements, were absent in the SmDDA1b promoter of E31 compared with E32 (Fig. S2B, see online supplementary material), this phenomenon was conserved in another four resistant and six susceptible materials (Fig S2D, see online supplementary material). The qRT-PCR results showed that *SmDDA1b* had high transcript accumulation in the leaves of both E31 (R) and E32 (S) plants, but a lower expression in the root, stem, and leaf of E32 plants compared with E31 (Fig. 2A; FigsS3 and S4A, see online supplementary material). The SmDDA1b protein level was also higher in E31 stem and root than in E32 (Fig S4D, see online supplementary material). The *SmDDA1b* was downregulated both in E31 and E32 plants from 1 h to 12 h *R. solanacearum* inoculation. Notably, *SmDDA1b* expression increased drastically in E31 plants but remained reduced in E32 plants after 24 h *R. solanacearum* inoculation (Fig. 2B; Fig. S4B, see online supplementary material). *SmDDA1b* was also induced in E31 plants but suppressed in E32 plants after 48 h following treatment with exogenous SA (Fig. 2C; Fig. S4C, see online supplementary material). Thus, these results demonstrated that *SmDDA1b* might involve in BW resistance.

### *SmDDA1b* positively regulates BW resistance

To evaluate the function of *SmDDA1b* in BW resistance, we generated 10 lines of *SmDDA1b*-silenced plants from the BW-resistant line E31 by virus-induced gene silencing (VIGS) in eggplant. *SmDDA1b* expression was reduced in the *SmDDA1b*-silenced eggplant plants (pTRV2-*SmDDA1b*) compared to the control plants (pTRV2) (Fig. 2D). All *SmDDA1b*-silenced eggplant lines displayed typical wilt symptoms with a high disease index after inoculation with *R. solanacearum*, while the control plants showed no significant wilt symptoms (Fig. 2E; Fig.S5A, see online supplementary material). To further determine the function of *SmDDA1b*, we overexpressed *SmDDA1b* in BW-susceptible tomato plants. Seven independent transgenic tomato lines highly expressing *SmDDA1b* were obtained and self-crossed to produce another generation for seed propagation and phenotypic characterization (Fig. S5B and C, see online supplementary material). Three representative transgenic tomato lines (OET_1–2_ OET_1–4_ OET_1–8_) were selected from the new generation for further analysis (Fig. S5D, see online supplementary material). The WT tomato plants exhibited wilted phenotype 7 d after inoculation with *R. solanacearum*, while the transgenic tomato OET_1–2_ OET_1–4_ and OET_1–8_ lines only displayed slight wilt in several leaves (Fig. 2G; Fig.S5D, see online supplementary material). We also measured the dynamic disease index and morbidity of WT and OE-*SmDDA1b* transgenic tomato plants after 14 days of *R. solanacearum* inoculation. The result showed that transgenic tomato OET_1–2_ OET_1–4_ and OET_1–8_ lines invariably had lower disease index values and morbidity than the WT plants (Fig. 2H and I; Fig.S5E, Table S2, see online supplementary material). When 100 μM 1-aminobenzotriazole (ABT, a salicylic acid inhibitor) were pre-sprayed 24 h before *R. solanacearum* inoculation, the resistance of OET_1–4_ plants to BW was weakened (Fig S5F and G, see online supplementary material). The results indicated that SmDDA1b have positive role in regulating BW resistance.

### SmDDA1b inhibits the spread of *R. solanacearum*

Because self-reproduction and spread of *R. solanacearum* occur in the xylem of plants [3], to investigate the SmDDA1b resistance mechanism to *R. solanacearum*, we analysed *R. solanacearum* colonization in the root, lower stem, and upper stem of WT and transgenic plants after inoculation. Consistent with the BW-susceptible pTRV2-*SmDDA1b* eggplant plants, higher *in vivo R. solanacearum* concentrations were detected in the root, lower and upper stem of *SmDDA1b*-silenced eggplant plants compared with the control plants (Fig. 2J). Interestingly, when *pTRV2-SmDDA1b* eggplant plants were almost completely wilted (14 dpi), the bacterial concentrations in their lower stems were 10^7.42^ CFU/g, significantly higher than that after 1 dpi and 7 dpi (Fig. 2E and J). Conversely, the control plants (pTRV2) showed robustness with extremely low bacterial concentrations in stems. Low bacterial concentrations were also observed in the stems of *SmDDA1b*-overexpressing tomatoes (OET_1–4_ plants), which only showed minor wilting symptoms (Fig. 2G and K). However, the bacterial concentrations in the WT tomato stem increased over time after inoculation with severe wilting (Fig. 2G and K). These results indicated that *SmDDA1b* positively regulates BW resistance by inhibiting the spread of *R. solanacearum* within plants.

### SmDDA1b positively regulates SA content and signaling pathway

**Figure 3:**
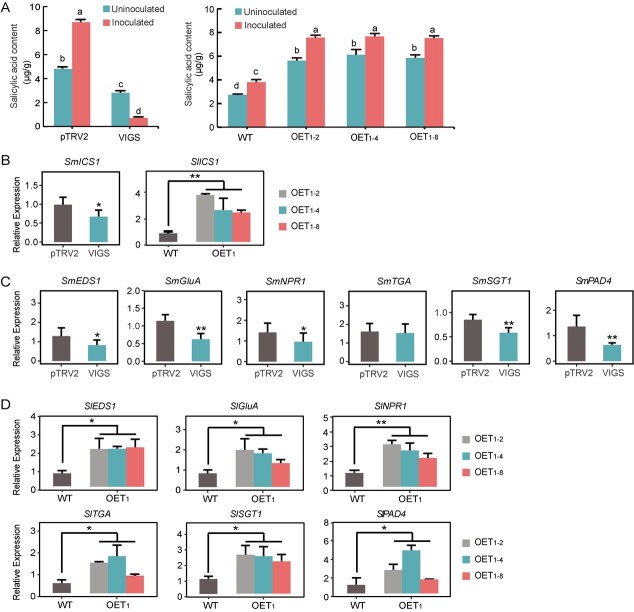
SmDDA1b-mediated positive regulation of SA content and signaling pathway. (**A**) The salicylic acid content of the control (pTRV2) and *SmDDA1b*-silenced eggplant plants (VIGS), the WT and *SmDDA1b*-overexpressing tomato seedlings with or without *Ralstonia solanacearum* inoculation. Samples (leaves) obtained 7 d after inoculation with *R. solanacearum* were used for analysis. Data are shown as mean ± SEM values of three biological replicates. Different letters indicate statistically significant differences among the groups (Tukey’s honest significant difference test, *P* < 0.05). (**B**) Expression of *ICS1* in *SmDDA1b*-silenced plants (VIGS) and OE-*SmDDA1b* plants. (**C**) Expression of SA signal pathway-related genes in the *SmDDA1b*-silenced and control plants. pTRV2 represents the control plants, while VIGS represents *SmDDA1b*-silenced plants. Data are shown as mean ± the SEM of three biological replicates (^*^P < 0.05; ^**^P < 0.01, Student’s *t*-test). (**D**) Expression of SA signal pathway-related genes (*SlEDS1, SlGluA, SlNPR1, SlTGA, SlSGT1,* and *SlPAD4*) in OE-*SmDDA1b* and the WT tomato plants. OET1 represents the T1 generation overexpression plants, including OET1–2, OET1–4, and OET1–8 lines. Data are expressed as mean ± the SEM of three biological replicates (^*^P < 0.05; ^**^P < 0.01, Student’s *t*-test). The reference gene was *SmActin* in eggplant. The *SlActin* was used as control gene in tomato.

Considering the important role of SA in BW resistance, we analysed SA levels in transgenic plants. The SA contents were repressed in *SmDDA1b*-silencing eggplant plants compared with the control eggplants (pTRV2) (Fig. 3A) but elevated in *SmDDA1b*-overexpressing tomato plants (line OET_1–2_ OET_1–4_ OET_1–8_) compared to the WT tomato plants (Fig. 3A). Moreover, the level of SA increased in control plants but declined in the *SmDDA1b*-silenced eggplant plants after *R. solanacearum* inoculation (Fig. 3A). The results showed that *SmDDA1b* positively controls SA levels in plants. We also detected the expression of SA biosynthesis-(*ICS1*) and signaling pathway-related genes (*SmEDS1*, *SmGluA*, *SmNPR1*, *SmSGT1*, *SmPAD4*). *SmICS1*, *SmEDS1*, *SmGluA*, *SmNPR1*, *SmSGT1*, *SmPAD4* were decreased in the *SmDDA1b*-silenced eggplant plants compared with the control plants (Fig. 3B and C; Fig. S6, see online supplementary material). Contrarily, *SlICS1* and the SA signaling genes were upregulated in the OE-*SmDDA1b* tomato plants compared with the WT plants (Fig. 3B and D; Fig. S7, see online supplementary material). These results demonstrated that *SmDDA1b* positively regulates the SA pathway.

### SmDDA1b suppresses SmNAC protein level through degradation

**Figure 4:**
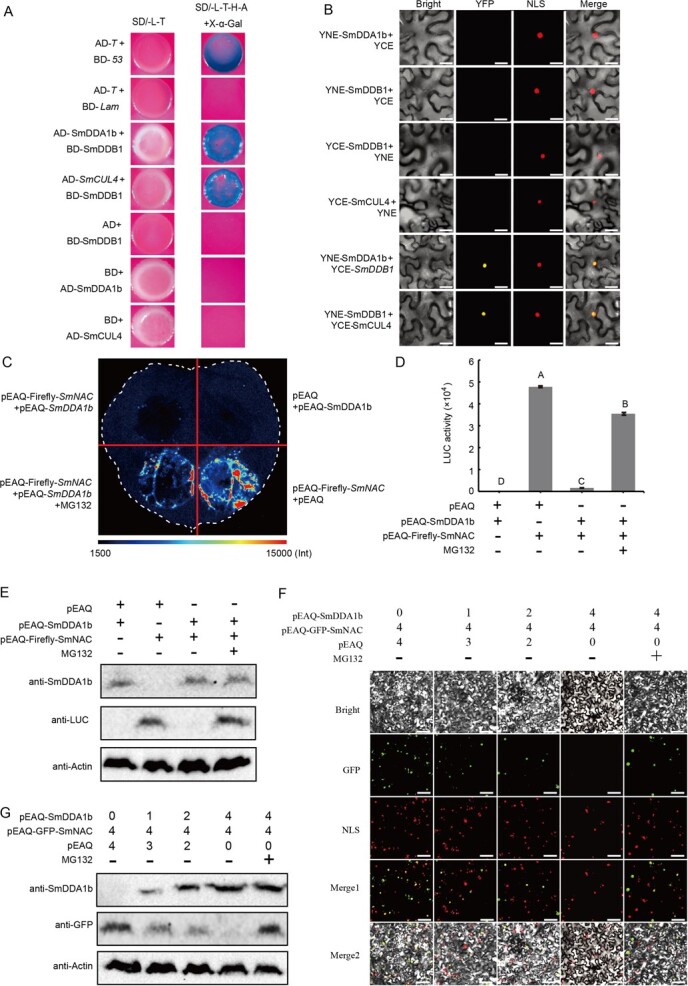
SmDDB1 interacted with both SmCUL4 and SmDDA1b and SmDDA1b degradation of SmNAC through the proteasome. (**A**) Y2H assays indicating the interaction of SmDDA1b with SmDDB1, SmCUL4 with SmDDB1. The AD-*T* and BD-*53* or BD-*Lam* co-transformed in the Y2H Gold strain was used as the positive or negative controls, respectively. (**B**) BiFC assays between SmDDA1b and SmDDB1, SmCUL4 and SmDDB1. Scale bars indicate 50 μm. (**C**) SmDDA1b-mediated proteasome degradation of SmNAC. For the four treatments of each tobacco leaf, the white dotted line indicates the outline of the tobacco leaf. The pEAQ-Firefly-SmNAC+pEAQ and pEAQ+pEAQ-SmDDA1b treatments were used as the positive and negative controls, respectively. MG132 is a proteasome inhibitor that inhibits protein degradation via the 26S proteasome. (**D**) The activity assay of firefly luciferase. The ‘+’ or ‘-’ symbol indicates a sample was added or omitted in each experiment, respectively. Data are expressed as mean ± SEM values of three biological replicates. (**E**) Western blot results. The ‘+’ or ‘-’ symbol indicates a sample was added or omitted in each experiment, respectively. Anti-SmDDA1b represents SmDDA1b protein antibody, anti-LUC represents Firefly protein antibody, and anti-Actin represents plant Actin protein antibody. (**F**) SmDDA1b-mediated proteome degradation of SmNAC visualized via Merge 1 and Merge 2. Different numbers represent different injection ratios. The ‘+’ or ‘-’ symbol indicates a sample was added or omitted in each experiment, respectively. NLS indicates the nucleus localization, while Merge 1 indicates the imaging combination of NLS and GFP. Merge 2 represents the combination of all the above images. The scale bar indicates 1 mm. (**G**) Western blot results. Different numbers represent different injection ratios. The ‘+’ or ‘-’ symbol indicates a sample was added or omitted in each experiment, respectively. Anti-GFP represents GFP protein antibody.

Because SmDDA1b is a CRL4 substrate receptor, we tested the interaction between SmDDB1 and SmDDA1b or SmCUL4. The Y2H and BiFC assays indicated that SmDDB1 have interaction with both SmCUL4 and SmDDA1b (Fig. 4A and B), implying a possible ubiquitin ligase role of SmDDA1b in eggplants. SmDDA1b also interacted with SmNAC in the nucleus (Fig. 1B). After the proteasome inhibitor MG132 [25] treatment, YFP fluorescence signal increased in the nucleus (Fig. 1B), suggesting that SmDDA1b may interact with SmNAC in the nucleus.

**Figure 5:**
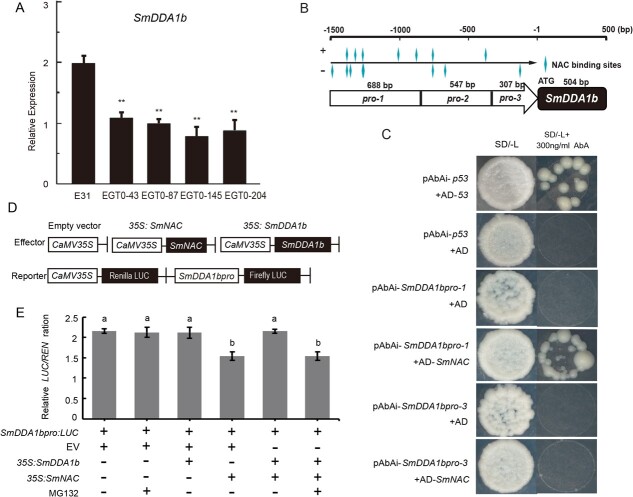
The binding of SmNAC to the *SmDDA1b* promoter represses *SmDDA1b* expression. (**A**) The accumulation of *SmDDA1b* in SmNAC over-expressed (OE-SmNAC) lines. E31 indicates wild-type, and EGT0–43, EGT0–87, EGT0–145, EGT0–204 represent T0 generation OE-*SmNAC* plants. Data are indicated as mean ± SEM values of three biological replicates [**P < 0.01, two-way analysis of variance (ANOVA)]. (**B**–**C**) Y1H assays between SmNAC and the *SmDDA1b* promoter. The AD-53 and pAbAi-p53 co-transformed in the yeast cells (Y1H Gold) served as the positive control, while the co-transformed pAbAi-p53 and AD were used as the negative control. (**D**–**E**) The repression of the *SmDDA1b* promoter by SmNAC. The regulation of promoter activity was according to the ratio of LUC to REN. The ‘+’ or ‘-’ symbols indicate a sample added or omitted in each experiment, respectively. EV indicates an empty vector, while MG132 is the proteasome inhibitor that inhibits protein degradation through the 26S proteasome. Data are expressed as mean ± SEM values of five biological replicates. Different letters indicate statistically significant differences among the groups (Tukey’s honest significant difference test, *P* < 0.05).

To further confirm whether SmDDA1b degraded SmNAC through the 26S proteasome, we performed degradation assay *in vivo*. The pEAQ-Firefly-SmNAC tobacco leaves showed normal firefly fluorescence signals, while weakened after infiltration with pEAQ-SmDDA1b (Fig. 4C). However, when the proteasome inhibitor MG132 was co-infiltrated with the pEAQ-Firefly-SmNAC and pEAQ-SmDDA1b, the firefly fluorescence signal increased again (Fig. 4C). The firefly luciferase activity also exhibited the same patterns (Fig. 4D). The Western blot (WB) results showed that when pEAQ-SmDDA1b and pEAQ-Firefly-SmNAC were co-infiltrated into the tobacco leaf, only SmDDA1b protein bands were displayed (Fig. 4E). However, when the proteasome inhibitor MG132 was co-infiltrated, SmNAC protein bands (anti-LUC) were appearing, which demonstrated that SmDDA1b degrade SmNAC protein by the 26S proteasome (Fig. 4E). Additional *in vivo* degradation assays were performed with different mixture ratios of the solutions of recombinant plasmid carrying GFP protein. The SmNAC-GFP fluorescence signal significantly weakened when the concentration of SmDD1b was increased (Fig. 4F). However, the addition of MG132 enhanced the GFP fluorescence signal of SmNAC-GFP (Fig. 4F). The results of WB showed that with the increase of SmDDA1b protein level, the level of SmNAC protein decreased (Fig. 4G). However, after the addition of MG132, the level of SmNAC protein increased. Those results show that SmDDA1b could inhibit the SmNAC protein level through degradation.

### SmNAC binds to the *SmDDA1b* promoter to repress *SmDDA1b* transcription

*SmDDA1b* expression was decreased in the OE-*SmNAC* plants compared with the WT plants [24] (Fig. 5A), suggesting *SmDDA1b* suppression by SmNAC. We found 24 NAC element binding sites in the *SmDDA1b* promoter obtained from the BW-resistant eggplant line E31, and the sites were mostly distributed in the −500 to −1500 region of the initiation codon (Fig. 5B; Table S3, see online supplementary material). For yeast one-hybrid (Y1H) analysis, the promoter of *SmDDA1b* was divided into three segments: *SmDDA1bpro-1* (−1542 to −855), *SmDDA1bpro-2* (−854 to −308), and *SmDDA1bpro-3* (−307 to −1) due to the self-activation of the full-length of the *SmDDA1b* promoter (Fig. 5B). *SmDDA1bpro-1* and *SmDDA1bpro-3* were used to perform interaction assays, as *SmDDA1bpro-2* was self-activating (Fig. S8, see online supplementary material). The results indicated that SmNAC directly binds to *SmDDA1bpro-1* (Fig. 5C). Moreover, the dual-luciferase assay showed that SmNAC repressed *SmDDA1b* transcription (Fig. 5D-E; S8C). When the mixture *Agrobacterium tumefaciens* solution containing *35S:SmDDA1b*, *35S:SmNAC*, and *SmDDA1bpro:LUC* constructs were co-infiltrated into *N. benthamiana*, the reduced effect of SmNAC on *SmDDA1* disappeared (Fig. 5E). However, this depressor of SmNAC on the *SmDDA1b* was recovered after co-infiltration with the proteasome inhibitor MG132 (Fig. 5E). Thus, SmNAC could bind the *SmDDA1b* promoter and significantly repress *SmDDA1b* transcriptional expression, and in turn, SmDDA1b degrade SmNAC by the UPS.

## Discussion

DDA1 has been widely studied in *Arabidopsis* (referred to as AtDDA1 in the present work) [16] and rice (OsDDA1) [26]. DDA1b negatively regulates the endogenous ABA-mediated developmental responses in plants. DDA1 can also interact with COP10 to inhibit photomorphogenesis [15]. However, a few studies have been reported on the involvement of DDA1 in regulating the SA pathway. The present study found that the *Arabidopsis* AtDDA1 and eggplant SmDDA1b are evolutionarily distant (Fig. S1A, see online supplementary material), and high homology proteins of SmDDA1b have not been studied. We also found that SmDDA1b targeted SmNAC for degradation through the UPS, thus positively regulating the SA pathway and BW resistance. Thus, our study enriches the current understanding of the function of CRL4 E3 ubiquitin ligase and emphasizes the significance of the UPS in regulating the SA pathway and defense responses.

A lot of E3 ubiquitin ligases participate in plant disease resistance. For example, E3 ubiquitin ligases MIEL1 and GhPUB17 have a negative role in defense responses in *Arabidopsis* [27] and cotton (*Gossypium* spp.) [28], respectively. However, E3 ligase NbUbE3R1 and PUB4 have positive roles in immune responses in tobacco [29] and *Arabidopsis* [30], respectively. E3 ligase NtRNF217 and the ATL family gene *StACRE* have positive roles in BW resistance in tobacco [31] and potatoes [32], respectively. Our study found that *SmDDA1b* expression was significantly induced by both *R. solanacearum* and SA treatment (Fig. 2B and C; Fig. S4B and C, see online supplementary material), with the expression pattern resembling the pattern triggered immunity (PTI) and effector triggered immunity (ETI) in plant disease resistance (reviewed in [33] and [34]).

*SmDDA1b* was not expressed in BW-susceptible eggplants (E32) after 24 h inoculation with *R. solanacearum* or after 48 h SA treatment (Fig. 2B and C; Fig. S4B and C, see online supplementary material). Based on the difference expression of *SmDDA1b* in the BW-resistant and susceptible materials after treatment with *R. solanacearum* and SA, we hypothesized that *SmDDA1b* regulates the BW resistance via the SA pathway in eggplants. Indeed, *SmDDA1b* silencing plants showed reduced the BW resistance. The SA contents, and *ICS1* and SA pathway signaling-related genes expression also deceased in *SmDDA1b* silencing plants. In contrast, *SmDDA1b* overexpression plants indicated increased BW resistance. The SA content, *ICS1* and SA pathway signaling-related genes expression also increased in *SmDDA1b* overexpression plants (Fig. 3; FigsS6 and S7, see online supplementary material). Thus, these results supported the hypothesis that *SmDDA1b* positively regulates BW resistance in an SA-dependent manner. The results further highlight the complexity and precision of the SA signaling pathway and disease resistance regulatory networks in plants.

CRL E3 ubiquitin ligase regulated the expression of SA pathway signaling genes. In *Arabidopsis*, CRL3 recognizes and degrades the SA pathway gene NPRs [35, 36]. In addition, the constitutive degradation of NPR3 monomers by CRL1 leads to preventing autoimmunity without the threat of pathogens [37]. HOS15, a substrate receptor of CRL1, interacts and degrades NPR1; additionally, and NPR1 may interact with CRL4 E3 ligase in *Arabidopsis*. In this study, the SA pathway genes, such as NPR1, show differential expression in the silenced and overexpression of *SmDDA1* plants (Fig. 3C and D; Figs S6B and D,S7B, see online supplementary material). Beside regulating SA synthesis by *ICS1*, the possible interaction between SA pathway signal genes and CRL4 E3 ligase, and the mutual regulation between CRLs, may be one reason for balancing SA pathway under normal environment and biotic stress. NAC transcription factors control gene expression and also associate with SA signaling; for example, the expression of *ONAC122*, *ONAC131* [38], *CaNAC035* [39], and *StNACb4* [9] can be induced by SA. SA is generally considered as a major plant hormone associated with disease resistance, such as bacterial wilt (BW). Similar to endogenous SA, exogenous SA can also enhance BW resistance [40]. In our previous study, SmNAC reduces BW resistance in eggplant by repressing the SA synthesis gene *ICS1* [24].

NAC can also interact with E3 ubiquitin ligases. For example, SINAT5 ubiquitinate AtNAC which is a RING-type E3 ligase [41], and SINA recognizes and degrades NAC1 in tomatoes through the UPS [42]. In this study, we found that ubiquitin ligase SmDDA1b interacts with SmNAC (Fig. 1A and B). Previous studies hypothesized that DDA1 acts as a substrate receptor for the multi-subunit E3 ligase CRL4, promoting the target protein recognition by CRL4 [15]. We confirmed that SmDDA1b is a component of CRL4 (Fig. 4A and B) and a homolog of AtDDA1 [16] (Fig. S1 and Table S1, see online supplementary material); thus, SmDDA1b can be reasonably inferred to act as a substrate receptor for CRL4. E3 ubiquitin ligase has specificity in recognizing target proteins; thus, to identify the target proteins are critical for dissecting the function of E3 ubiquitin ligases. We found that SmDDA1b can interact with its target protein SmNAC (Fig. 1A and B). Moreover, the target protein recognized by E3 ubiquitin ligase is degraded by 26S proteasome [11]. In addition, it is interesting to observe that SmNAC targeted the SmDDA1b promotor and repressed its expression (Fig. 5). The ability of NAC binds to the E3 promoter has also been reported in other studies. In banana, MaNAC1 and MaNAC2 directly binding to the promoter of MaXB3 and repress its expression [43].

**Figure 6:**
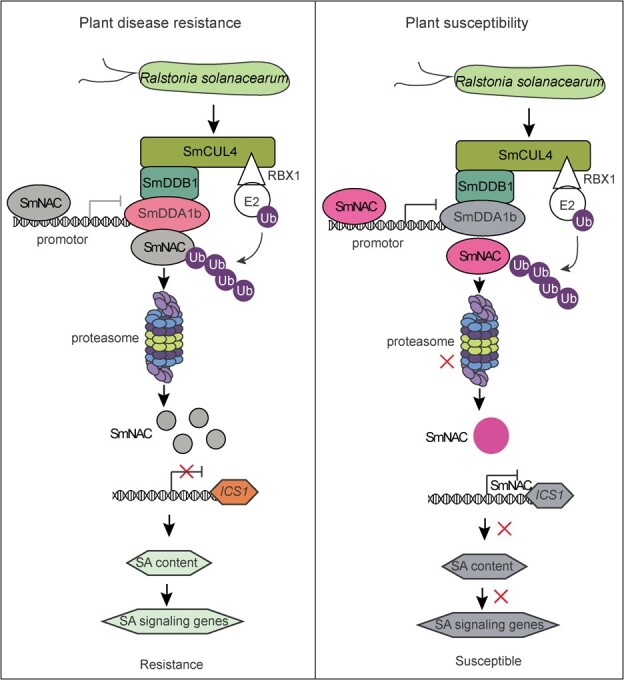
The SmDDA1b regulatory module enhances plant resistance to BW. For disease resistance plants, during *Ralstonia solanacearum* stress, the SmDDA1b proteins were induced by *R. solanacearum*. SmNAC were recognized by SmDDA1b and then degraded by the SmDDA1b-mediated ubiquitin/26S proteasome system (UPS). Consequently, the feedback regulatory of SmNAC on *SmDDA1b* were a failure and the suppression of SmNAC on *ICS1* was also relieved, SA is accumulated and the SA signaling genes are activated, thus system-acquired resistance (SAR) is induced in plants. For susceptible plants, during *R. solanacearum* stress, SmDDA1b proteins were restrained, SmNAC cannot be recognized and degraded by ubiquitin/26S proteasome system (UPS). The released SmNAC proteins inhibits the expression of *SmDDA1b* in return and the suppression of SmNAC on *SmICS1* was enhanced, SA and SA signaling pathway was repressed.

In general, all the results support the hypothesis that SmDDA1b can improve the BW resistance of eggplants by SmNAC-mediated SA pathway. For disease resistance plants, during *R. solanacearum* stress, the SmDDA1b proteins were induced by *R. solanacearum*. SmNAC were recognized by SmDDA1b and then degraded by the SmDDA1b-mediated ubiquitin/26S proteasome system (UPS). Consequently, the feedback regulatory of SmNAC on *SmDDA1b* were a failure and the suppression of SmNAC on *ICS1* was also relieved, SA is accumulated and the SA signaling genes are activated, thus system-acquired resistance (SAR) is induced in plants. For susceptible plants, during *R. solanacearum* stress, SmDDA1b proteins were restrained, SmNAC cannot be recognized and degraded by the ubiquitin/26S proteasome system (UPS). The released SmNAC proteins inhibits the expression of *SmDDA1b* in return and the suppression of SmNAC on *SmICS1* was enhanced, SA and SA signaling pathway was repressed (Fig 6). Similar molecular regulatory patterns have also been reported in other species. In *Populus*, PalWRKY77 was degraded by U-box E3 ligase PalPUB79 and PalWRKY77 directly represses PalPUB79 transcription [44]. In Tartary buckwheat (*Fagopyrum tataricum*), FtMYB11 was targeted by E3 ligase FtBPM3, and FtMYB11 also repress *FtBPM3* expression [45]. And in banana, the RING type E3 ligase MaXB2 is responsible for degrading transcription factors MaNAC2 and MaNAC3, as well as ethylene biosynthesis proteins MaACS1 and MaACO3. Simultaneously, MaNAC2 and MaNAC3 act to inhibit the expression of MaXB2 [43], indicating a feedback regulatory mechanism between these genes that helps maintain a balance of gene expression levels.

The molecular mechanism of *SmDDA1b* regulating eggplant resistance to BW ultimately boils down to the regulation of the SA pathway. Therefore, *SmDDA1b* may also have other functions, such as regulating plant cold stress resistance. SA has been proven to alleviate and regulate various physiological and biochemical changes in plants caused by cold stress [46–48]. Therefore, it is speculated that cold stress may induce *SmDDA1b* expression, which leads to an increase in the expression of genes related to the SA pathway and thus resistance to cold stress.

Our study identified 22, 34, 42, 89, 49, 31, and 27 putative NAC elements in the promoters of *SmGluA*, *SmNPR1*, *SmPAD4*, *SmSGT1*, *SmTGA*, *SmEDS1*, *ICS1*, respectively (Tables S4–S9, see online supplementary material). Previous studies have shown that NAC transcription factors bind the promoters of *ICS1*, *EDS1*, *PAD4* [49]. SmNAC may also directly bind the promoters of SA pathway signaling-related genes. However, further clarifications on whether SmNAC directly binds to SA pathway-related genes are necessary.

## Materials and methods

### Experimental materials

BW-resistant E31 (R) and BW-susceptible E32 (S), two inbred lines of eggplants (*S. melongena*) were used in this study (Fig.S9 and Table S10, see online supplementary material). *Nicotiana benthamiana*, *S. lycopersicum* cultivar ‘Money Maker’, and *R. solanacearum* strain GMI1000 were also used in the study.

### Gene expression analysis

For plant total RNA isolation and complementary DNA (cDNA) synthesis, the Promega RNA extraction kit (Promega, Shuanghai, China) and the EZB reverse transcription kit (EZBioscience, Roseville, MN, USA) were used. For qRT-PCR, the Vazyme mix (Vazyme, Nanjing, China) was used. The qRT-PCR primers are listed in Table S11 (see online supplementary material). The reference genes used in eggplant were SmActin and SmCyclophilin. The reference genes used in tomato were SlActin and SlGAPDH.

### Yeast two-hybrid assay

*SmDDA1b* and *SmCUL4* CDS sequences were constructed into the pGADT7 vector. Thereafter, the N-terminal of *SmNAC* (1–139 aa) and the full-length *SmDDB1* ORF which removed the stop codon, were cloned into the pGBKT7 vector. The specific primers are shown in Table S12 (see online supplementary material). The experiment was performed based on the manufacturer’s instructions (Cat. No. 630489; Clontech, Mountain View, CA, USA).

### Bimolecular fluorescence complementation analysis

The CDS sequences of SmDDA1b, *SmDDB1*, and *SmCUL4* without the stop codons were cloned into the pSPYNE-35 s/pUC-SPYNE (YNE) vector. The residue genes without the stop codons were constructed into the pSPYCE-35 s/pUC-SPYCE (YCE) vector. *A. tumefaciens* GV3101 (pSoup) with the construct, mixed with *A. tumefaciens* cells harboring DsRed protein (*v*:*v*:*v*, 1:1:1), and infiltrated into *N. benthamiana* leaves. Proteasome inhibitor MG132 (50 μM) was also infiltrated into *N. benthamiana* leaves and the plants were cultivated in the dark at 22°C for 3 d. The GFP fluorescence was visualized by confocal fluorescence microscope (Carl Zeiss, Oberkochen, Germany). The assays were repeated three times. The primers used are listed in Table S12 (see online supplementary material).

### Coimmunoprecipitation assay

SmDDA1b and SmNAC CDS sequences were introduced into a plant transient expression vector pEAQ with a GFP tag for SmDDA1b, His tag for SmNAC. *A. tumefaciens* GV3101 strains contained the indicated constructs were mixed. Then, the mixture infiltrated into leaves of *N. benthamiana* plants at the 4-week-old stage. After 36 h and 48 h incubation, the infiltrated leaf tissues were used to extract proteins with extraction buffer [50 mM HEPES (pH 7.5), 150 mM KCl, 1 mM EDTA, 1 mM DTT, 0.5% TritonX-100, and protease inhibitor cocktail (Sigma-Aldrich)], combined with 30 μL Protein A + G Agarose (Beyotime, catalog #P2012) and 2 μL GFP antibody (abcam, catalog #ab290), and incubated overnight at 4°C. 1 × PBS buffer and was used to wash the Agarose beads, elution buffer [200 mM glycine (pH = 2.5) and 1 M Tris base (pH = 10.4)] was used to elute. The eluate proteins were used immediately or stored at −80°C. Before the SDS-PAGE and IB analysis, 1 × SDS loading buffer was mixed into the samples and then boiled for 10 min.

### Subcellular localization analysis

*SmDDA1b* CDS sequence without stop codons was constructed into the pEAQ-EGFP vector, and then introduced into *A. tumefaciens* strain GV3101(pSoup). *A. tumefaciens* cells containing DsRed protein (*v*:*v*, 1:1) mixture infected *N. benthamiana* leaves. The plants were cultivated in the dark at 22°C for 3 d. A confocal fluorescence microscope (Carl Zeiss, Oberkochen, Germany) was used to detect the green fluorescent protein (GFP) fluorescence. The assays were repeated three times. The primers used are listed in Table S12 (see online supplementary material).

### Phylogenetic analysis and sequence alignment

DDA1-containing sequences from15 dicotyledonous plants were acquired by blasting the whole-genome protein sequences (Table S1, see online supplementary material) in the NCBI RefSeq database using Hmmserch v3.3. Thereafter, the sequences were aligned using the “—auto” parameter of Mafft v7.455 software, and visualized by DNAMAN. The phylogenetic tree was built by default parameters of Iqtree v1.6.12.

### Data analysis

2^-△ct^ and 2^-△△ct^ were processed in Excel. Student’s *t*-test, Tukey’s honest significant difference test and two-way analysis of variance (ANOVA) were processed by IBM SPSS Statistics 20 using 0.01 or 0.05 significance levels. Data are expressed as mean ± SEM or mean ± SD.

### Pathogen inoculation

*R. solanacearum* inoculation was performed according to our previous study [23]. The experiment was conducted in three biological replicates under controlled conditions (30°C during 16 h of light and 24°C during 8 h of dark), and the leaf samples that collected at 0 h, 1 h, 3 h, 6 h, 12 h, and 24 h were used to perform qRT-PCR analysis.

### Hormonal treatment

Eggplant seedlings at the four-leaf stage were sprayed with 1 mM of SA every 12 h for two days, spraying SA until all leaves of the plant were covered with hormone droplets [40, 50]. Water treatment was control. The plants were cultivated under normal conditions (26°C during 16 h of light and 22°C during 8 h of dark). For qRT-PCR analysis, leaf samples were used from three biological replicates at 0 h, 3 h, 6 h, 12 h, 24 h, and 48 h after SA treatment.

### Virus-induced gene silencing assays

A 300 bp fragment of *SmDDA1b* was constructed into the pTRV2 vector. pTRV1, pTRV2, and pTRV2-*SmDDA1b* vectors were infected the *A. tumefaciens* strain GV3101. A mixture of pTRV1 and pTRV2 or pTRV2-SmDDA1b (*v*:*v*, 1:1) infiltrated into the leaves of eggplant seedlings at four- or five-leaf-old stage. The plants were maintained at 16°C in the dark for 1 d, then all the plants were cultivated under normal conditions for one to two weeks (26°C during 16 h of light, 22°C during 8 h of darkness). There were 10 biological replicates for each treatment. The primers used are shown in Table S12 (see online supplementary material).

### *SmDDA1b* overexpression vector construction and transformation process

The full-length CDS of *SmDDA1b* was amplified and joined into the pCAMBIA-1380 vector. The *Agrobacterium* strain GV3101 with pCAMBIA-1380-*SmDDA1b* overexpression vector was transformed into the tomato cultivar ‘Money Marker’ [51].

### Extraction of total plant protein and Western blot assay

Plant protein extraction kit (Solarbio, BC3720) was used to obtain total plant protein. Refer to [52] for the specific steps of western blot assay. The anti-SmDDA1b, anti-LUC antibody, and anti-GFP antibody were used for *in vivo* ubiquitination assay. The peptide sequence selected for the SmDDA1b antibody was: MEDTSSSIPPNNATTSGAAKYLAGLPSRGLFSSNVLSSTPGGMRVYICDHETSPPEDQFIKTNQQNILIRSLMLKKQRGDHSSKDGKGISSNDNGRKRAAEKTLDSRTSNKKATTSNQVASPQETSRIRTPDIQNMTVEKLRALLKEKGLSLRGRKDELIARLRGDT, and the catalog numbers of anti-Actin antibody, anti-LUC antibody, and anti-GFP antibody were AB_764433, AB_934495, and AB_950071, respectively.

### Salicylic acid extraction and quantification

Leaves of *SmDDA1b*-silenced plants, control plants, *SmDDA1b*-overexpressing lines, and WT before and after inoculation with *R. solanacearum* were collected for SA extraction and quantification [53, 54]. The catalog number for the standard SA was 69–72-7 (Tianjin Damao Chemical Reagent Co. Ltd, Tianjin, China).

### Effect of SA biosynthesis inhibitor on *R. solanacearum* resistance of *SmDDA1b*-overexpressing plants

The WT and *SmDDA1b*-overexpressing plants at four-leaf stage were prespayed with 100 μM 1-aminobenzotriazole (ABT, a salicylic acid inhibitor) 24 h before being inoculated with *R. solanacearum. R. solanacearum* inoculation was performed according to our previous study [23].

### *R. solanacearum* isolation and quantification

Whole eggplants inoculated with GMI1000 were collected after 1, 7, and 14 d of inoculation with *R. solanacearum*. The roots, lower stems and the upper stems were successively washed. The samples were soaked in 75% ethanol for 30 s and washed twice with sterile water (ddH_2_O) under sterile conditions. The homogenized samples by sterile quartz sand and ddH_2_O were filled with ddH_2_O to 10 mL in a 50 mL tubes. The solution was then diluted to a 10^1^–10^6^ gradient series. 100 μL of each dilution was spread on TTC medium containing 50 mg/L rifampicin. The cells were counted after incubation at 30°C for 2 d. The *A. tumefaciens* was not grown at this time on the TTC plate (Fig. S10, see online supplementary material) and PCR was additionally used to confirm the identity of putative *R. solanacearum* isolates (Table S12, see online supplementary material). At least three biological replicates per treatment.

### *In vivo* degradation

The CDS sequence of the SmNAC that removing the stop codon was constructed into pEAQ-Firefly and pEAQ-GFP vectors, while CDS sequence of the SmDDA1b that removing the stop codon was constructed into the PEAQ vector. The *A. tumefaciens* GV3101 with recombinant plasmids were infected *N. benthamiana* seedlings leaves at 6- or 7-leaf-old stage. Four groups of the recombinant *A. tumefaciens* mixture were injected into four different parts of each leaf: Group 1: pEAQ+pEAQ-SmDDA1b (*v*:*v*, 1:1); Group 2: pEAQ-Firefly-SmNAC+pEAQ (*v*:*v*, 1:1); Group 3: pEAQ-Firefly-SmNAC+pEAQ-SmDDA1b (*v*:*v*, 1:1); Group 4: pEAQ-Firefly-SmNAC+pEAQ-SmDDA1b + MG132 (*v*:*v*:*v*, 1:1:1). For Group 4, MG132 was infiltrated where the *A. tumefaciens* culture had been infiltrated for 36 h–48 h, and firefly luciferase substrate was subsequently infiltrated 3 d after MG132 infiltration. The chemiluminescence imager was used to observe the leaf luminescence (Bio-Rad/ChemiDoc XRS+, USA), and an enzyme-labeling instrument (Biotek/Cytation 5, Winooski, VT, USA) was used to detect firefly luciferase activity.

The mixed *Agrobacterium* cells carrying pEAQ-GFP-SmNAC and pEAQ-SmDDA1b were infected *N. benthamiana* leaves. The amount of pEAQ-GFP-SmNAC infiltrated into the leaves was fixed, while that of pEAQ-SmDDA1b was gradually increased. The ratio of infiltration is, respectively, pEAQ SmDDA1b: pEAQ GFP SmNAC = 0, 0.25, 0.5, and 1. After 36 h–48 h of the treatment, the same amounts of pEAQ-GFP-SmNAC, pEAQ-SmDDA1b, and MG132 were injected into the treatment group. The luminescence was then observed by fluorescence microscope 3 d after the second treatment.

### Promoter isolation and element prediction

The *SmDDA1b* gene was found by TBtools v1.09852 from eggplant v4 genome(https://solgenomics.net). PlantPAN 3.0 (http://plantpan.itps.ncku.edu.tw) was used to detect the NAC element in the 1543-bp promoter. The primers used are shown in Table S12 (see online supplementary material).

### Yeast one-hybrid assay (Y1H)

*SmNAC* CDS sequence was joined into the pGADT7 vector, *SmDDA1b* promoter sequence was constructed into the pAbAi vector. The Y1H assay was performed based on the manufacturer’s protocol (Clontech, USA). The used primers are listed in Table S12 (see online supplementary material).

### Dual-luciferase assay

pGreen II 0800-LUC vector-SmDDA1b-promoter was used as a reporter, empty pGreenII 62-SK, pGreenII 62-SK-*SmDDA1b*, and pGreenII 62-SK-*SmNAC* were used as effectors. The *N. benthamiana* leaves were infected by the *A. tumefaciens* strain GV3101 containing effector and reporter constructs (*v*:*v*, 20:1). After 24–36 h incubation, MG132 (50 μM) was subsequently infiltrated into the leaves. Dual-Luciferase Reporter Gene Assay Kit (Yeasen, Shanghai, China) and Cytation 5 Cell Imaging Multi-Mode Reader (BioTek, Winooski, VT, USA) were used to measure firefly LUC and Renilla LUC activities. The primers used are listed in Table S12 (see online supplementary material).

## Acknowledgements

We thank Chuhao Li (South China Agricultural University) for his help in drawing figures and Lianhui Zhang (South China Agricultural University) for providing the GMI1000 strain.

This research was funded by the Key R&D Projects in Guangdong Province (2022B0202080003), the Key Project of Guangzhou (202103000085), the seed industry revitalization project of Guangdong (2022NPY00026), Fruit and Vegetable Industry System Innovation Team Project of Guangdong (2021KJ110), and the National Natural Science Foundation of China (31672156).

## Author contributions

S.Y., Y.W., and B.Y. performed the research; B.C., Z.Q., and J.L. designed the research; C.C., Y.G., and Z.Z. provided new reagents; and S.Y., Y.W., Z.Q., and B.C. wrote the manuscript.

## Data availability

All relevant data generated or analysed are included in the manuscript and the supporting materials.

## Conflict of interest statement

The authors have no conflicts of interest.

## Accession numbers

The GenBank/Sol Genomics Network (SGN) accession numbers of the sequences reported in this paper are as follows: SmNAC, KM435267; SmDDA1b, MZ736671; DDB1, SMEL_002g158220.1.01; CUL4, SMEL_002g157970.1.01; ICS1, SMEL_006g263050.1.01; SmEDS1, SMEL_006g263300.1.01; SmGluA, SMEL_001g150160.1.01; SmNPR1, SMEL _000g071090.1.01; SmSGT1, SMEL_006g251310.1.01; SmPAD4, SMEL_002g157190.1.01; SmTGA, SMEL_010g361040.1.01; SlICS1, NM_001247865.2; SlEDS1, NM_001320249.1; SlGluA, M80604.1; SlNPR1, NM_001247633.1; SlTGA NM_001324613.1; SlSGT1, EF011105.1; SlPAD4, XM_004231563.4.

## Supplementary data

Supplementary data is available at *Horticulture Research* online.

## Supplementary Material

Web_Material_uhad246
