# Supplementary material for: A putative E3 ubiquitin ligase substrate receptor degrades transcription factor SmNAC to enhance bacterial wilt resistance in eggplant
Source: Hortic Res. 2023 Nov 27;11(1):uhad246. doi: 10.1093/hr/uhad246 (PMC10794948; doi:10.1093/hr/uhad246)
Supplement: Web_Material_uhad246 [file web_material_uhad246.pdf]

Supporting Information

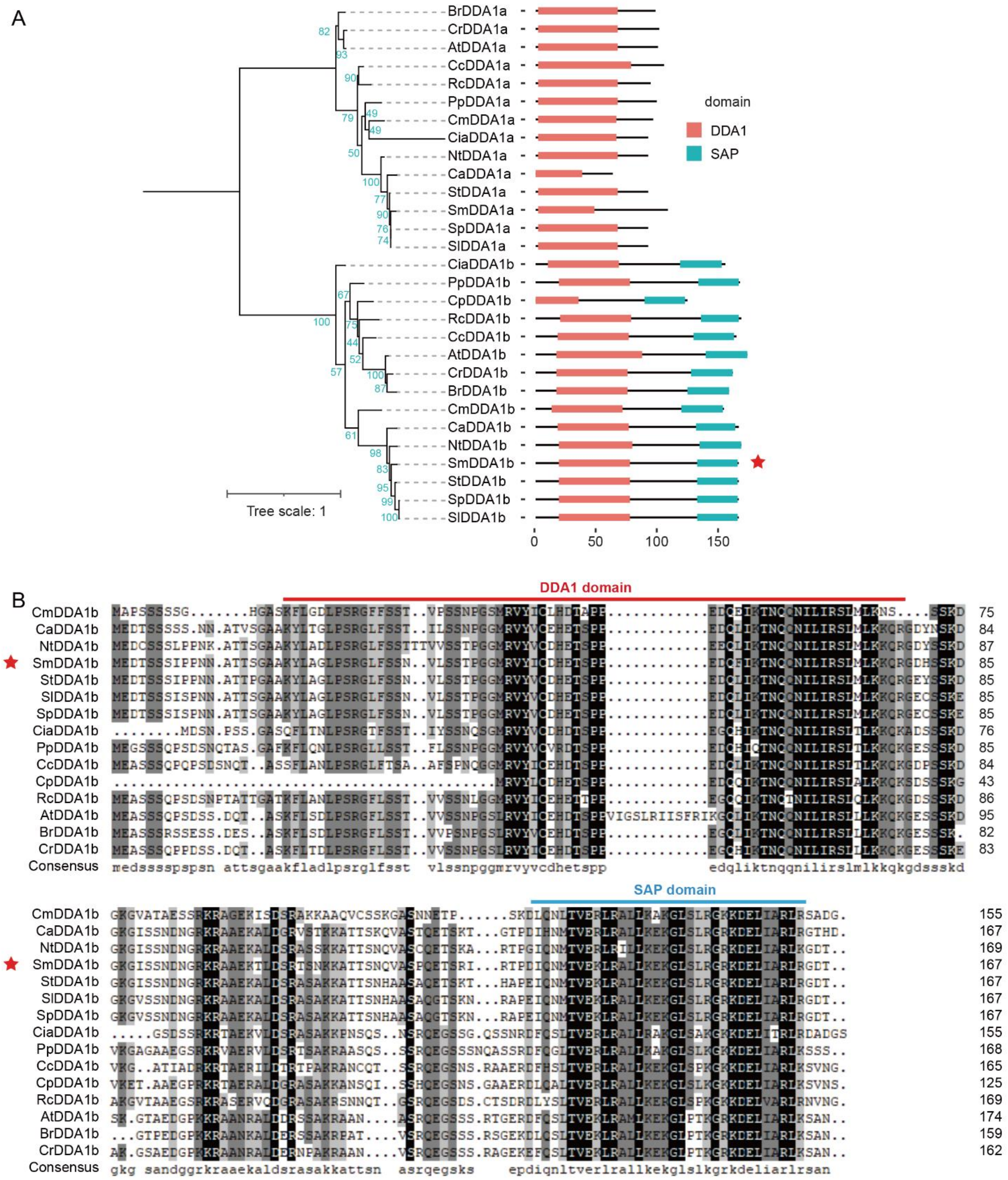

Figure S1. SmDDA1b phylogenetic and protein analysis.

(A) SmDDA1b phylogenetic analysis. HMMER was used to search the protein sequences of several species via the PF10172 model and '--cut_nc' as the filter threshold. The number on each branch represents the bootstrap support of the node to its right, out of a maximum value of 100. SmDDA1b is

indicated with a red star. The accession numbers of the genes are shown in Table S1. The red cylinder represents the DDA1 domain, and the green one represents the SAP domain. (B) Sequence alignment of SmDDA1b homologous proteins. The conserved DDA1 and SAP domains are indicated with red and green boxes, respectively. The red star indicates SmDDA1b.

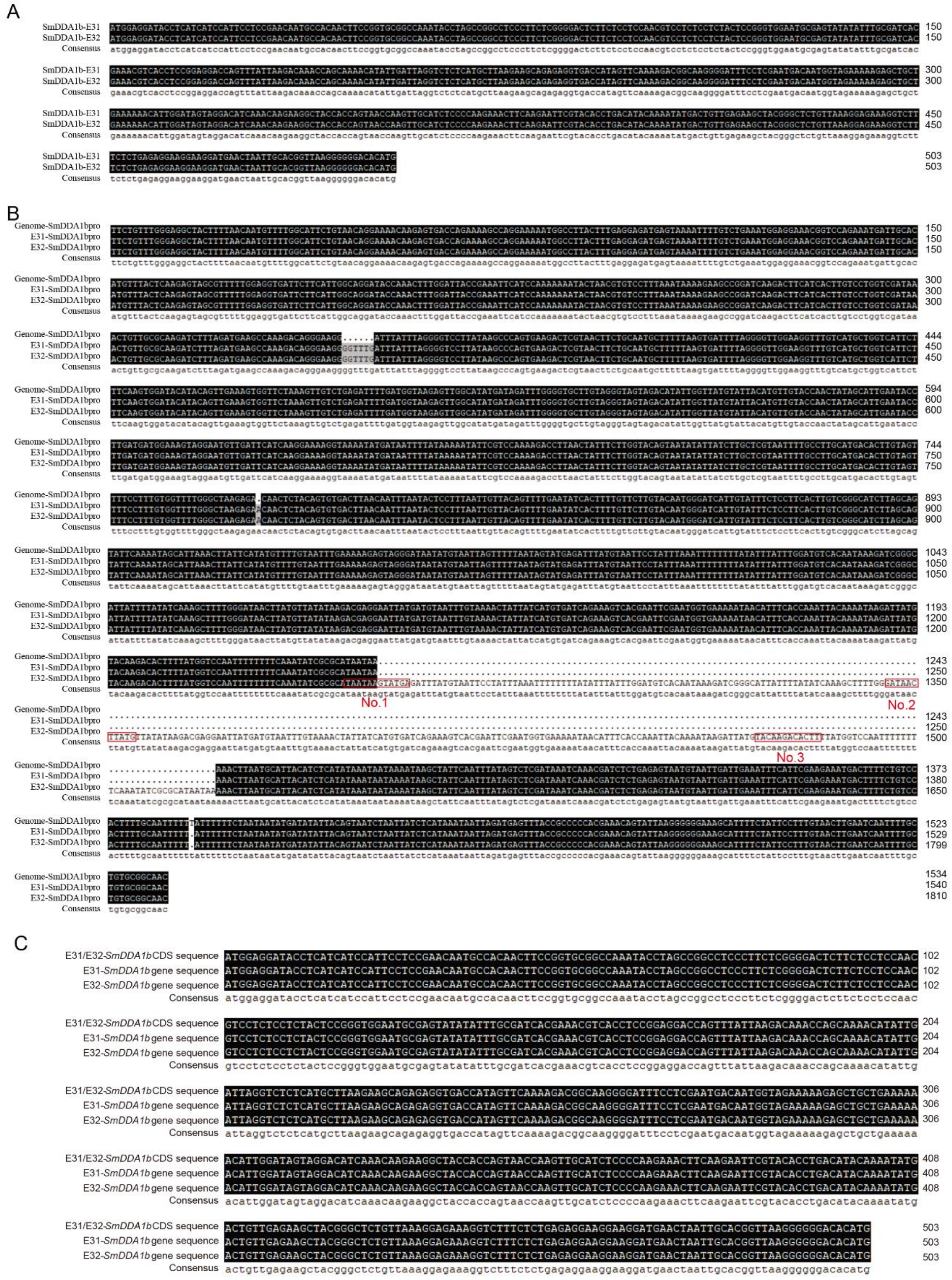

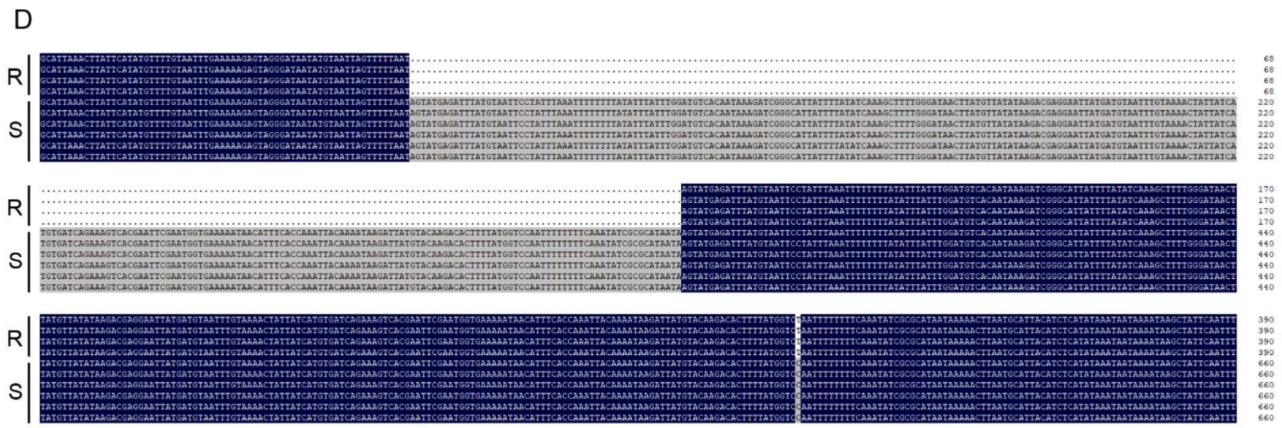

Figure S2. Comparison of *SmDDA1b* cDNA, promoter sequences and genome DNA sequences in E31 and E32.

(A) Comparison of *SmDDA1b* cDNA sequences in E31 and E32. No difference in *SmDDA1b* cDNA sequence was detected between the BW resistant line E31 and susceptible line E32. (B) Comparison of *SmDDA1b* promoter sequences in E31 and E32. No.1 to No.3 represented the NAC binding cis-acting element. (C) Comparison of *SmDDA1b* genome DNA sequences in E31 and E32. (D) Comparison of *SmDDA1b* promoter sequences in four resistance eggplants and 6 susceptible eggplants.

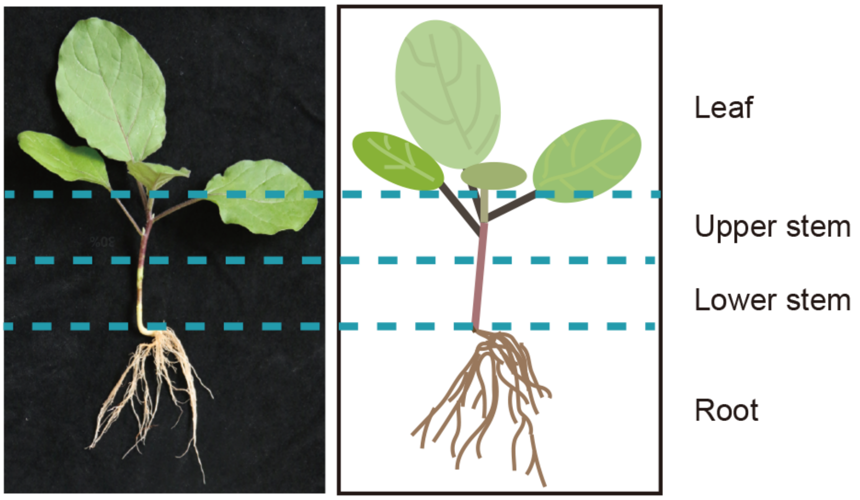

Figure S3. A schematic diagram of the tissue parts of eggplant seedlings (leaves, upper and lower parts of the stems, and roots).

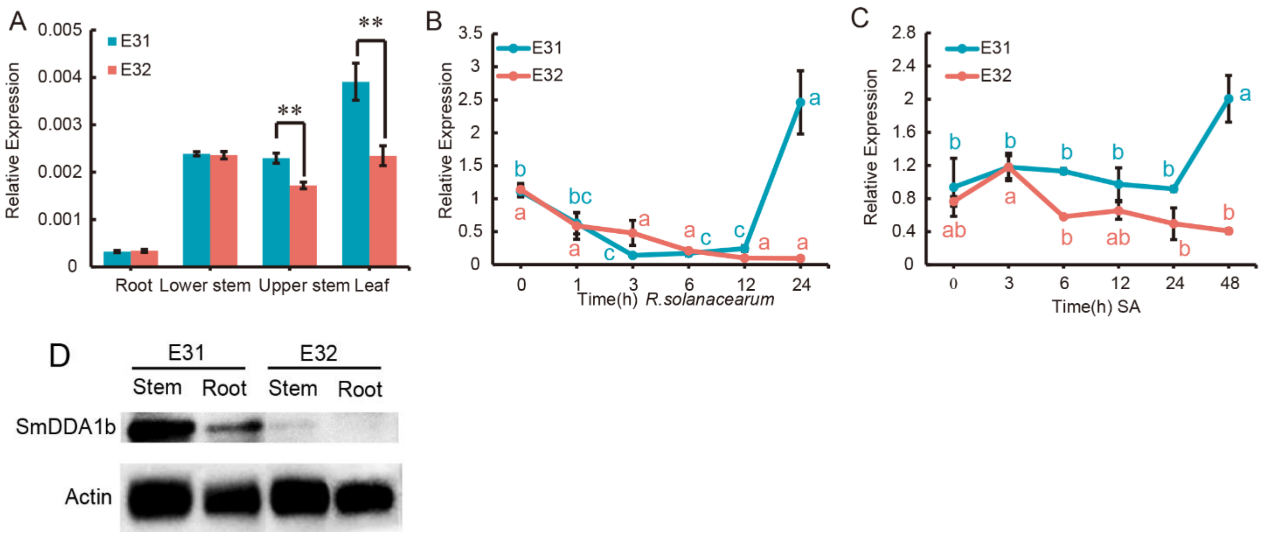

Figure S4. Expression pattern analysis of *SmDDA1b*. (A) Relative expression of *SmDDA1b* in E31 and E32 tissues. The relative expression was estimated using the $2^{-\Delta ct}$ method. The bar graph shows the relative expression of *SmDDA1b* in the roots, upper stem, lower stem, and leaves of E31 and E32 eggplants. Data are expressed as mean ± SD values ($n = 3$) (*, $p < 0.05$; **, $p < 0.01$, according to the Student's *t*-test). (B) Relative expression of *SmDDA1b* in E31 and E32 after inoculation with *R. solanacearum*. The samples (leaves) were obtained at 0 h, 1 h, 3 h, 6 h, 12 h, and 24 h after infection. (C) Relative expression of *SmDDA1b* in E31 and E32 after treatment with salicylic acid. The samples (leaves) were obtained at 0 h, 3 h, 6 h, 12 h, 24h, and 48 h after treatment. The relative expression was estimated using the $2^{-\Delta\Delta ct}$ method. Data are expressed as mean ± SEM values of the three biological replicates. Different letters indicate statistically significant differences among the groups (Tukey's honest significant difference test, $p < 0.05$). The *SmCyclophilin* was used as reference gene. (D) Western blot of SmDDA1b in stem and root of E31 and E32 plants.

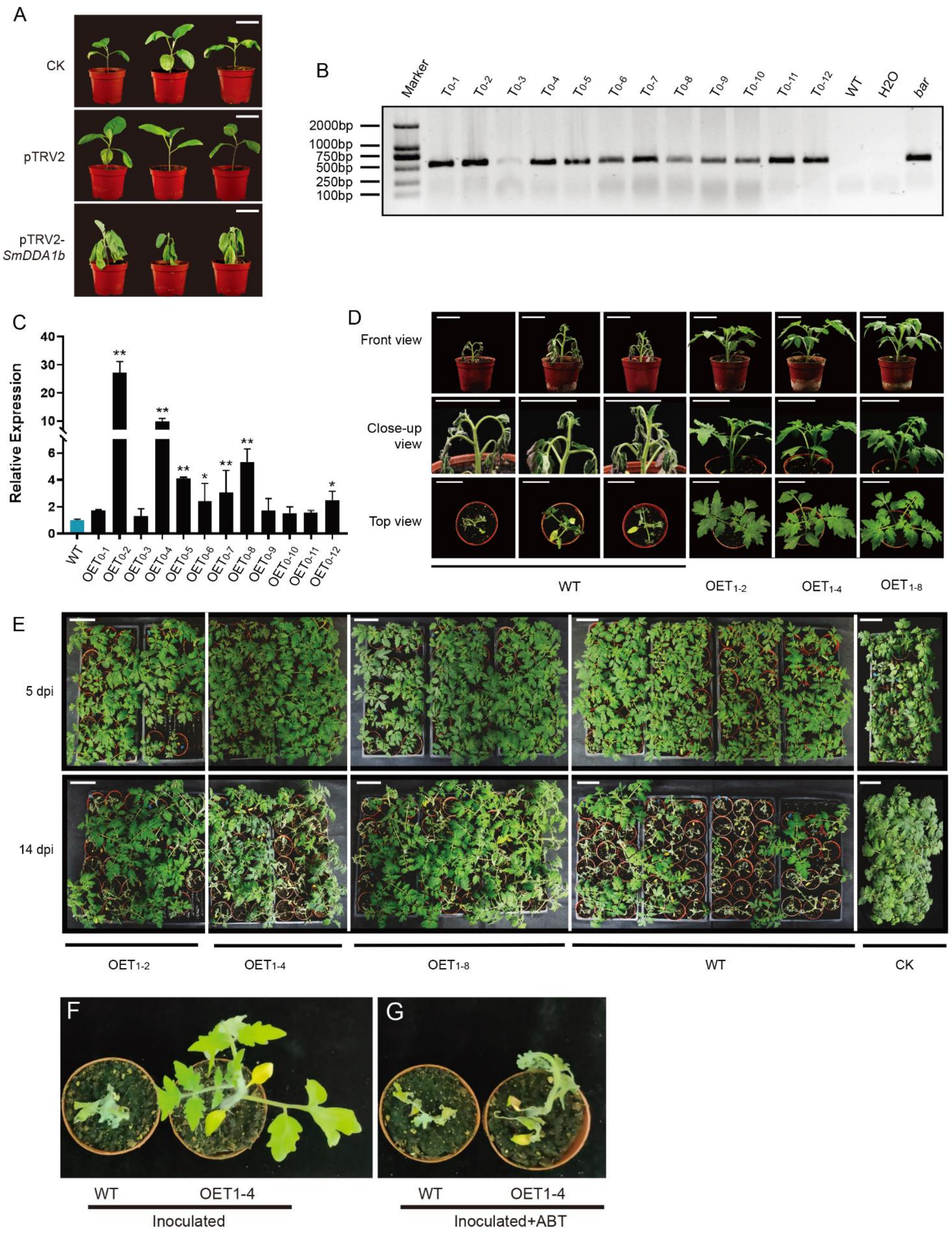

Figure S5. Identification of *SmDDA1b*-overexpressing plants in T_0 generation and the phenotype of *SmDDA1b*-silenced plant and *SmDDA1b*-overexpression plants after inoculated by *Ralstonia*

solanacearum. (A) The phenotype of the control and *SmDDA1b*-silenced plants 10 d after inoculation with *R. solanacearum*. CK and pTRV2 indicates the control group, and the plants were treated with water and empty vector infection solution, respectively. The pTRV2-*SmDDA1b* indicates virus induced gene silencing (VIGS)-treated plants. Scale bars indicate 5 cm. (B) Detection of the marker gene *bar* on tomato transgenic seedlings. (C) The relative expression of *SmDDA1b* in leaves of independent transgenic lines. The calculation methods using $2^{-\triangle\triangle ct}$. Data are expressed as mean ± SD (n=3) (*, $p < 0.05$; **, $p < 0.01$, according to Student's t-test). (D) The phenotype of WT and T_1 generation seedlings (OET_1), including the front view, close-up view, and top view of seedlings 7 d after inoculation with *R. solanacearum*. Scale bars indicate 5 cm. (E) The phenotype of WT, $OET_{1\text{-}2}$, $OET_{1\text{-}4}$, and $OET_{1\text{-}8}$ plants on the 5 days post inoculation (dpi) (at this time, the plant has not developed disease, as a control) and 14 dpi with *R. Solanacearum*. CK represents WT plants that were not inoculated with *R. Solanacearum*. Scale bar indicates 10 cm. (F-G) The WT and OE SmDDA1b (OET1-4) plants were presprayed with 100 µM 1-aminobenzotriazole (ABT, a salicylic acid inhibitor) 24h before inoculated with *R. solanacearum*.

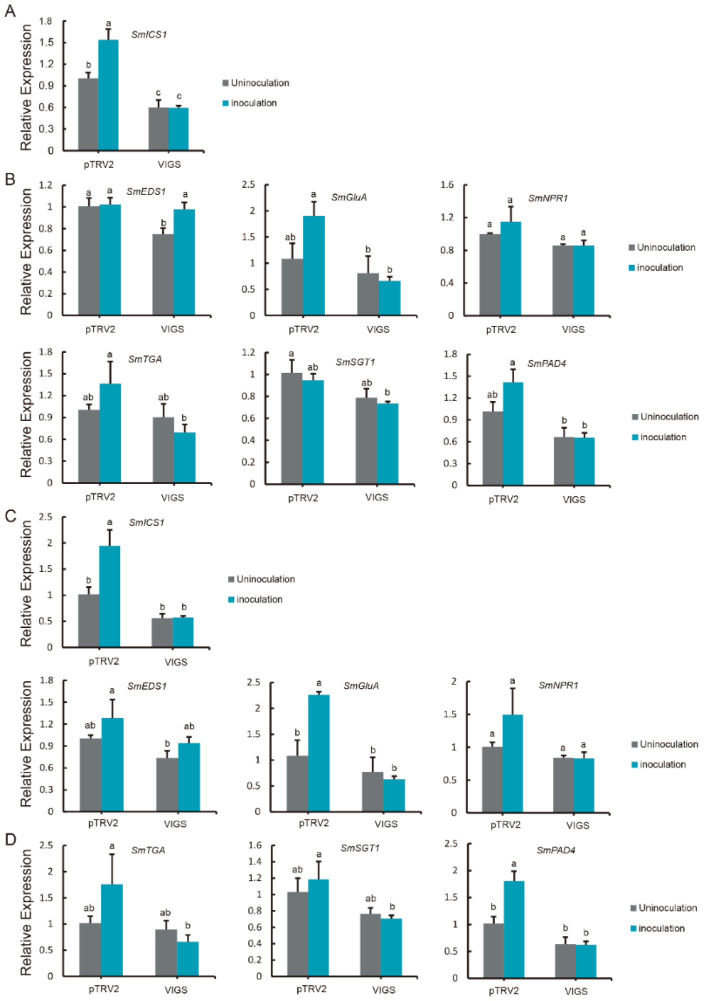

Figure S6. Expression of *ICS1*and SA pathway genes in *SmDDA1b*-silenced eggplant plants (VIGS) and control plants under uninoculation and inoculation condition. pTRV2 represents the control eggplant plants, while VIGS represents SmDDA1b-silenced plants. The relative expression was determined using the using $2^{-\triangle\triangle ct}$ method. Data are expressed as mean ± the SEM of three biological replicates (*, $p < 0.05$; **, $p < 0.01$, Student's *t*-test). The *SmActin* was used as reference gene in A-B, the *SmCyclophilin* was used as reference gene in C-D.

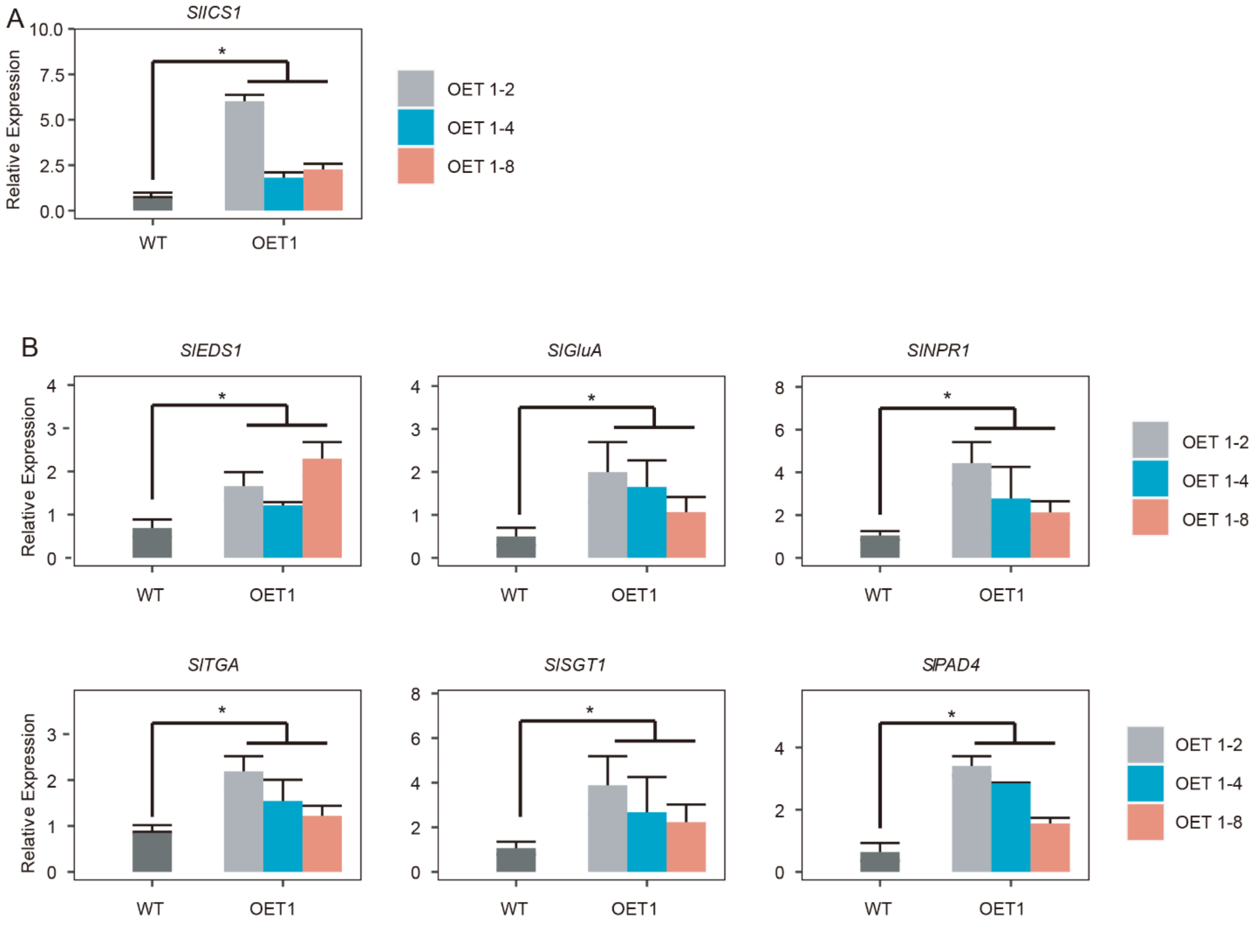

Figure S7. Expression pattern analysis of *SlICS1* and SA pathway genes in *SmDDA1b*-overexpression plants inoculated with *Ralstonia solanacearum*. Expression of *SlICS1* (A) and SA signal pathway-related genes (*SlEDS1, SlGluA, SlNPR1, SlTGA, SlSGT1,* and *SlPAD4*) (B) in OE-*SmDDA1b* and the WT plants. OET1 represents the T1 generation overexpression plants, including OET_{1-2}, OET_{1-4}, and OET_{1-8} lines. The relative expression was calculated using the $2^{-\triangle\triangle ct}$ method. Data are expressed as mean ± the SEM of three biological replicates (*, $p < 0.05$; **, $p < 0.01$, Student's *t*-test). The *SlGAPDH* was used as control gene.

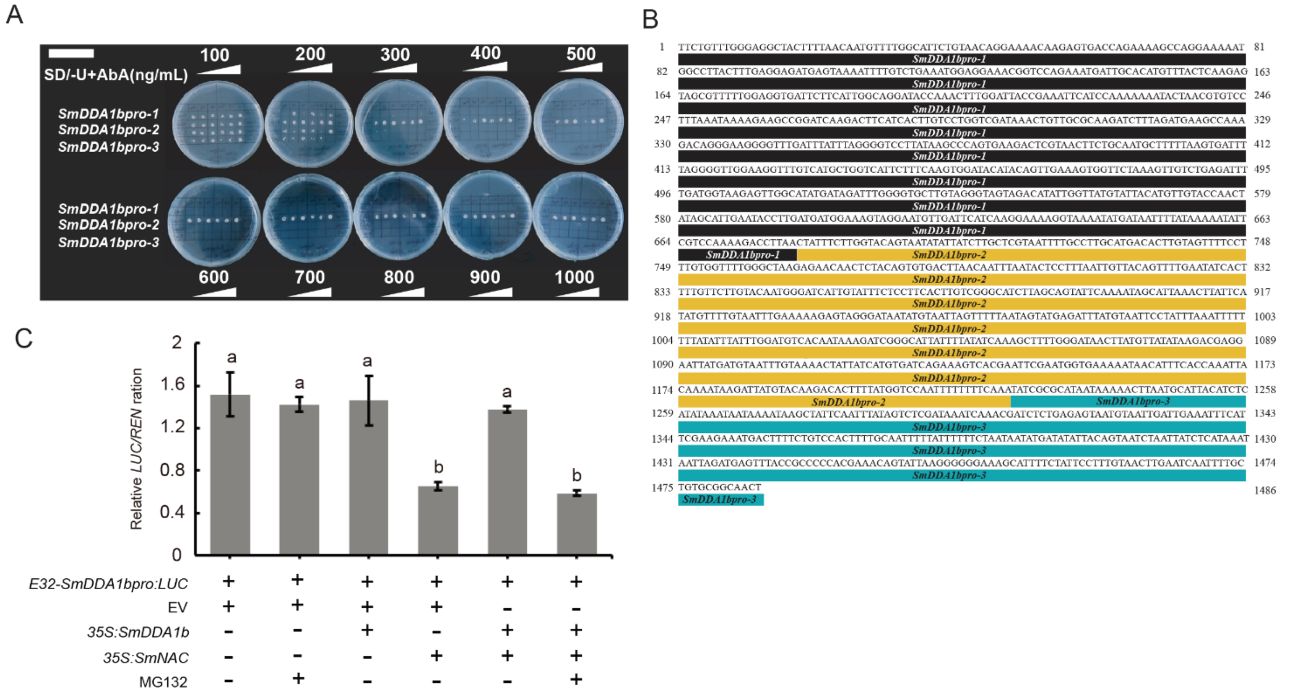

Figure S8. The *SmDDA1bpro-2* has self -activation and repression of the *SmDDA1b* promoter by SmNAC. (A) *SmDDA1bpro-2* has self-activation. The self-activation of *SmDDA1bpro-1*, *SmDDA1bpro-2,* and *SmDDA1bpro-3* was detected. After the recombinant vector was transferred into Y1H gold, it was spotted on Ura-deficient medium (SD/-U) with different AbA concentrations (ng/ml). The number represents the AbA concentration, the triangle indicates the yeast concentration from low to high. (B) The promoter of *SmDDA1b* was divided into three segments for Y1H assay. *SmDDA1bpro-1*: -855 to -1542 bp, *SmDDA1bpro-2*: -308 to -854 bp, *SmDDA1bpro-3*: -1 to -307 bp. Different segmented areas are distinguished by different colors. (C) The repression of the *SmDDA1b* promoter by SmNAC reflected by LUC/REN assays.

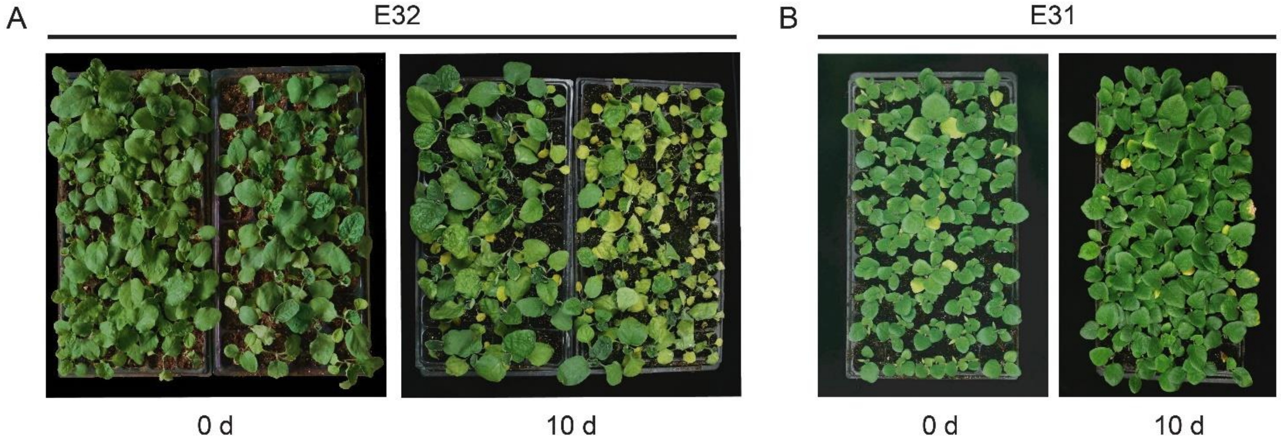

Figure S9. Detection of disease resistance of E31 and E32 to *Ralstonia solanacearum*. Growth of E32

and E31 on 0 d and 10 d after inoculation with *R. solanacearum* (GMI1000). The size of the plug in the figure is 5 × 10, 50 holes in total. E32 showed obvious wilting phenotype, while E31 was not significantly altered at 10 days after inoculated. For specific data refer to Table S10.

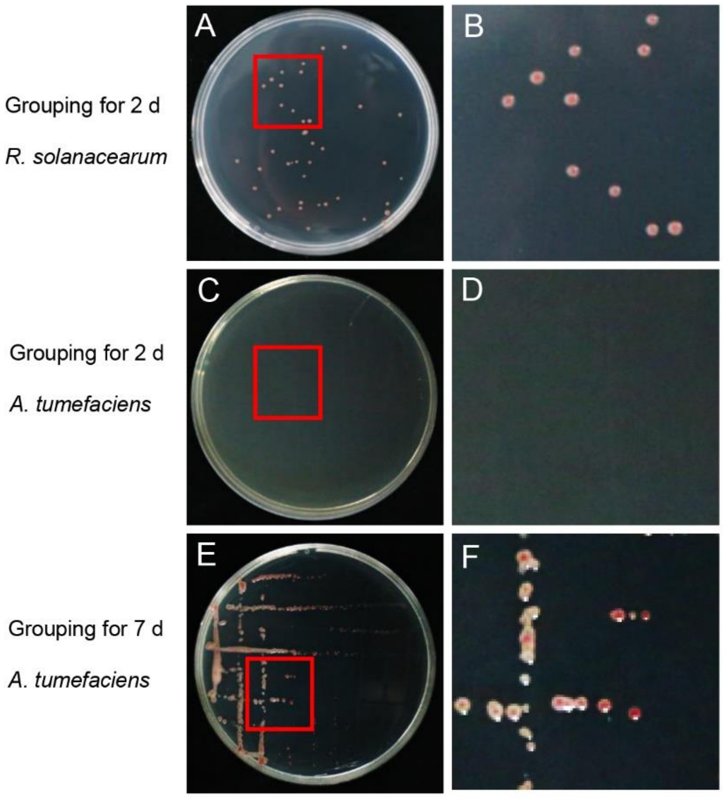

Figure S10. The colonies of *A. tumefaciens* and *R. solanacearum* on the TTC plate.
(A-B) the *R. solanacearum* clonies after culture 2 days on the TTC plate. (C-F) the *A. tumefaciens* clonies after culture 2 days (C-D) and 7 days (E-F) on the TTC plate.

Supporting Information

Table S1 A statistical table of 15 species and their genome accession numbers used in the *SmDDA1b* phylogenetic tree.

Gene	Species	Gene Accession
StDDA1b	*Solanum tuberosum*	XP_006351476.1
NtDDA1b	*Nicotiana tabacum*	XP_016475719.1
SpDDA1b	*Solanum pennellii*	XP_015067941.1
CaDDA1b	*Capsicum annuum*	XP_016563825.1
SlDDA1b	*Solanum lycopersicum*	XP_004236334.1
AtDDA1b	*Arabidopsis thaliana*	NP_001190606.1
CrDDA1b	*Capsella rubella*	XP_006281225.1
BrDDA1b	*Brassica rapa*	XP_009112080.1
CpDDA1b	*Carica papaya*	XP_021905455.1
CcDDA1b	*Citrus clementina*	XP_006440104.1
PpDDA1b	*Prunus persica*	XP_007209709.1
CmDDA1b	*Cucumis melo*	XP_008464442.1
CiaDDA1b	*Cicer arietinum*	XP_004515818.1
RcDDA1b	*Ricinus communis*	XP_015577745.1
SmDDA1b	*Solanum melongena*	Unigene0058253
AtDDA1a	*Arabidopsis thaliana*	NP_198971.1
CrDDA1a	*Capsella rubella*	XP_023634841.1
BrDDA1a	*Brassica rapa*	XP_009140096.1
RcDDA1a	*Ricinus communis*	XP_002509861.2
CcDDA1a	*Citrus clementina*	XP_024042665.1
PpDDA1a	*Prunus persica*	XP_020420462.1
CiaDDA1a	*Cicer arietinum*	XP_012569271.1
CmDDA1a	*Cucumis melo*	XP_008437335.1
NtDDA1a	*Nicotiana tabacum*	XP_016454173.1
CaaDDA1a	*Capsicum annuum*	XP_016552274.1

StDDA1a	*Solanum tuberosum*	XP_006352266.1
SlDDA1a	*Solanum lycopersicum*	XP_004244619.1
SpDDA1a	*Solanum pennellii*	XP_015083817.1
SmDDA1a	*Solanum melongena*	Unigene0022527

Table S2 List of plant morbidity and disease index in overexpression experiment.

Material	Time (d)	Total	Diseased plants	Healthy plants	Incidence (%)	Evaluation of scale					Disease index
						0	1	2	3	4	
WT	1	66	0	66	0	66	0	0	0	0	0
	2	66	0	66	0	66	0	0	0	0	0
	3	66	0	66	0	66	0	0	0	0	0
	4	66	0	66	0	66	0	0	0	0	0
	5	66	0	66	0	66	0	0	0	0	0
	6	66	9	57	13.63	57	5	2	1	1	6.06
	7	66	29	37	43.93	37	9	6	5	8	25.76
	8	66	40	26	60.61	26	5	8	10	17	45.08
	9	66	50	16	75.76	16	6	5	7	32	62.50
	10	66	52	14	78.79	14	2	3	5	42	72.35
	11	66	54	12	81.82	12	5	2	2	45	73.86
	12	66	54	12	81.82	12	4	3	2	45	74.24
	13	66	54	12	81.82	12	4	2	2	46	75.00
	14	66	54	12	81.82	12	4	2	2	46	75.00
OET_1	1	115	0	115	0	115	0	0	0	0	0.00
	2	115	0	115	0	115	0	0	0	0	0.00
	3	115	0	115	0	115	0	0	0	0	0.00
	4	115	0	115	0	115	0	0	0	0	0.00
	5	115	0	115	0	115	0	0	0	0	0.00
	6	115	4	111	3.48	111	1	3	0	0	1.52
	7	115	16	99	13.91	99	10	2	3	1	5.87

	8	115	27	88	23.48	88	12	2	6	7	13.48
	9	115	42	73	36.52	73	7	7	11	17	26.52
	10	115	52	63	45.22	63	8	5	6	33	36.52
	11	115	57	58	49.57	58	7	6	6	38	41.09
	12	115	61	54	53.04	54	8	6	8	39	43.48
	13	115	67	48	58	48	12	6	5	44	46.74
	14	115	68	47	59.13	47	7	10	7	44	48.70
OET_{1-2}	1	27	0	27	0	27	0	0	0	0	0.00
	2	27	0	27	0	27	0	0	0	0	0.00
	3	27	0	27	0	27	0	0	0	0	0.00
	4	27	0	27	0	27	0	0	0	0	0.00
	5	27	0	27	0	27	0	0	0	0	0.00
	6	27	1	26	3.7	26	0	1	0	0	1.85
	7	27	4	23	14.81	23	2	1	1	0	6.48
	8	27	9	18	33.33	18	3	1	2	3	21.30
	9	27	11	16	40.74	16	3	0	2	6	30.56
	10	27	15	12	55.56	12	1	3	2	9	45.37
	11	27	15	12	55.56	12	1	2	3	9	46.30
	12	27	15	12	55.56	12	1	2	3	9	46.30
	13	27	17	10	62.96	10	3	2	1	11	50.00
	14	27	18	9	67	9	3	3	1	11	51.85
OET_{1-4}	1	44	0	44	0	44	0	0	0	0	0.00
	2	44	0	44	0	44	0	0	0	0	0.00
	3	44	0	44	0	44	0	0	0	0	0.00
	4	44	0	44	0	44	0	0	0	0	0.00
	5	44	0	44	0	44	0	0	0	0	0.00
	6	44	2	42	4.55	42	1	1	0	0	1.70
	7	44	7	37	15.91	37	5	1	1	0	5.68
	8	44	11	33	25	33	5	0	4	2	14.20

	9	44	19	25	43.18	25	2	5	6	6	30.68
	10	44	20	24	45.45	24	3	1	2	14	38.07
	11	44	24	20	54.55	20	2	4	1	17	46.02
	12	44	26	18	59.09	18	4	2	3	17	48.30
	13	44	27	17	61.36	17	4	1	2	20	52.27
	14	44	27	17	61.36	17	0	4	3	20	55.11
OET_{1-8}	1	44	0	44	0	44	0	0	0	0	0.00
	2	44	0	44	0	44	0	0	0	0	0.00
	3	44	0	44	0	44	0	0	0	0	0.00
	4	44	0	44	0	44	0	0	0	0	0.00
	5	44	0	44	0	44	0	0	0	0	0.00
	6	44	1	43	2.27	43	0	1	0	0	1.14
	7	44	5	39	11.36	39	3	0	1	1	5.68
	8	44	7	37	15.91	37	4	1	0	2	7.95
	9	44	12	32	27.27	32	2	2	3	5	19.89
	10	44	17	27	38.64	27	4	1	2	10	29.55
	11	44	18	26	40.91	26	4	0	2	12	32.95
	12	44	20	24	45.45	24	3	2	2	13	36.93
	13	44	23	21	54.55	21	5	3	2	13	39.20
	14	44	23	21	54.55	21	4	3	3	13	40.34

Table S3 List of NAC transcription factor binding sites.

Position	Strand	Sequence
121	+	atggaGGAAAcgg
121	+	atggAGGAAacgg
122	+	tggaGGAAAcggt
148	+	cACATG
184	+	TTCTTcattggcaggata
237	+	tactaACGTGtcctt

238	+	actaACGTGtcctttaaataa
239	+	ctaaCGTGTcctttaaataaa
494	+	ttgatGGTAAga
624	+	atcaaGGAAAagg
624	+	atcaAGGAAaagg
625	+	tcaaGGAAAaggt
741	+	tagtTTTCCtttg
13	-	ggctaCTTTTaacaatgtttt
122	-	tggaGGAAAcggt
238	-	actaACGTGtcctttaaataa
240	-	taaCGTGTcctttaaa
242	-	ACGTG
741	-	tagtTTTCCtttg
742	-	agtTTTCCtttgt
742	-	agttTTCCTttgt
828	-	tatcaCTTTTgttcttgtaca
1123	-	CATGTg
1370	-	gtccaCTTTTgcaatttttta

Table S4 List of NAC transcription factor binding sites in *SmGluA*.

Gene	*SmGluA*	
Position	Strand	Sequence
348	+	ataTACGTgacag
349	+	taTACGTgac
362	+	aacaaGGAAAaan
362	+	aacaAGGAAaaan
363	+	acaaGGAAAaann
1457	+	atttgCCGTGttg
1458	+	tttgcCGTGTtg

1834	+	ataTACGTgacag
1835	+	taTACGTgac
1848	+	aacaaGGAAAaat
1848	+	aacaAGGAAaaat
1849	+	acaaGGAAAaatg
351	-	TACGT
352	-	ACGTG
1327	-	ctttgCTTAAag
1414	-	aaTTGACct
1457	-	atttgCCGTGttg
1457	-	atttgCCGTGtt
1458	-	tttgcCGTGTtg
1837	-	TACGT
1838	-	ACGTG
1849	-	acaaGGAAAaatg

Table S5 List of NAC transcription factor binding sites in *ICS1* and *SmEDS1*.

Gene	*ICS1*		Gene	*SmEDS1*	
Position	Strand	Sequence	Position	Strand	Sequence
159	+	tttaaaaaatcAAAAGtactt	288	+	gagagGGTAAaa
224	+	aaGTCAAtt	299	+	atgtACGCAaacc
344	+	tacagcagACACGtaa	299	+	atgTACGCaaac
349	+	cagacACGTAa	300	+	tgtACGCAaa
350	+	agACACGtaaag	300	+	tgtACGCAaacc
350	+	agacACGTAaag	569	+	aTTACTtgt
351	+	gacACGTAaa	605	+	taGTCAAat
351	+	gACACGtaaaga	871	+	aagagGGTAAtt
351	+	gacACGTAaag	1006	+	tccACGTAaa
351	+	gacACGTAaagat	1006	+	tccACGTAaaatt

351	+	gACACGtaaag	1008	+	CACGT
352	+	acACGTAaaga	1009	+	ACGTA
353	+	CACGT	111	-	tcttaCTTTTatttttatttt
354	+	ACGTA	133	-	ctttgCTTTTtttttaaaaat
1050	+	tTACGTcgagttgaagt	164	-	ttttaCTTTTaaaaatacttt
1063	+	aaGTCAAgt	299	-	atgtACGCAa
1210	+	cagaaagttaaAAAAGtgtgg	299	-	atgtaCGCAAacc
1458	+	ttaaaaaaaaaAAAAGccact	299	-	atgTACGCaaac
1695	+	aggaACTTGttttcctaggaa	299	-	atgTACGCaaacc
1938	+	catTACGCatct	300	-	tgtACGCAaa
130	-	TACGT	300	-	tgTACGCaaa
349	-	cagacACGTAaag	301	-	gtaCGCAAac
351	-	gacACGTAaa	301	-	gtaCGCAAacc
352	-	ACACGtaaaga	301	-	gTACGCaaacc
1050	-	tTACGTcgagttgaagtca	339	-	actgTTTCCtgga
1051	-	TACGT	424	-	cACATG
1938	-	catTACGCatct	549	-	TACGT
			550	-	ACGTG
			713	-	atTTGACca
			937	-	aaatTCCTTacaa
			1073	-	ttatTCCTTatat

Table S6 List of NAC transcription factor binding sites in *SmNPR1*.

Gene	*SmNPR1*	
Position	Strand	Sequence
48	+	ataagtatttagCACGTcatc
237	+	gggaGGAAAaata
400	+	ttgTAAGTaataa
400	+	ttgtAAGTAataa

577	+	tcaaaacaaatAAAAGttaaa
605	+	caaaaaaaaaaAAAAGagagt
754	+	aaaACGTAaa
757	+	ACGTA
767	+	gatCACGGaatca
767	+	gatCACGGaatc
767	+	gatcACGGAatca
768	+	atCACGGaatca
778	+	caatgACGTGttcag
1330	+	ccACACGaaaaa
1331	+	cACACGaaaaat
1345	+	agtgaGGAAActt
1345	+	agtgAGGAAactt
1346	+	gtgaGGAAActtt
44	-	taaaataagtatttagCACGTc
55	-	tttagCACGTcatca
400	-	ttgTAAGTaataa
702	-	agtttCTTTTtctttttctaa
754	-	aaaACGTAaa
767	-	gatCACGGaatca
778	-	caatgaCGTGTt
778	-	caatGACGTgtt
779	-	aatgACGTGttcaggaccgaa
779	-	aatgaCGTGTtc
1332	-	ACACGaaaaat
1346	-	gtgaGGAAActtt
1407	-	aagcTTCCTtcag
1421	-	cACATG
1440	-	gttttCTTAAag

1573	-	tgTCTTGactgcaatg

Table S7 List of NAC transcription factor binding sites in *SmSGT1*.

Gene	*SmSGT1*		Gene	*SmSGT1*	
Position	Strand	Sequence	Position	Strand	Sequence
20	+	tatattaattgttACGTAa	29	-	tgtTACGTaatt
28	+	ttgtTACGTaat	29	-	tgTTACGtaatt
28	+	ttgttaCGTAAtt	29	-	tgTTACGtaattt
29	+	tgtTACGTaattt	30	-	gttACGTAat
29	+	tgTTACGtaatt	30	-	gtTACGTaat
29	+	tgttACGTAatt	30	-	gttACGTAattt
29	+	tgttaCGTAAtt	32	-	tACGTAat
30	+	gtTACGTaat	32	-	TACGT
30	+	gtTACGTa	101	-	attttACTTAtaa
30	+	gttACGTAat	537	-	tattttCGTGTt
30	+	gttACGTAatttt	538	-	attttCGTGTta
31	+	ttACGTAattt	538	-	attttCGTGTt
33	+	ACGTA	538	-	attttCGTGTta
101	+	attttACTTAtaa	540	-	tttCGTGTtaaagcta
103	+	tTTACTtat	605	-	tattaCTTTTaatttagtgga
521	+	taaataacataAAAAGtattt	646	-	tttcaCTTTTaacacctgttt
537	+	tattttCGTGT	791	-	tatgaCTTTTgatttattttt
537	+	tattttCGTGTt	957	-	acctaCTTTTaaattcttttt
606	+	aTTACTttt	967	-	aaattCTTTTtagcttttgga
666	+	tcactaatataAAAAGtagtc	987	-	ACGTG
762	+	aaGTCAAaa	1071	-	ccttaCTTTTtttcttttaag
950	+	tgGTCAAac	1108	-	gtTTGACta
1041	+	aaGTCAAaa	1122	-	tttagCTTTTtatctctaata
1611	+	gcactttggtatcAAGTTc	1171	-	tcctaCTTTTtttttaatatt

1657	+	cttttatcaaaaCACGTaact	1198	-	attttCTTTTtaaattttgtt
1660	+	ttatcaaaACACGtaa	1218	-	tctttCTTTTtttgttgctct
1665	+	aaaacaCGTAAct	1276	-	tctttCTTTTttttcttctaa
1666	+	aaACACGtaact	1611	-	gcactttggtatCAAGTtcat
1666	+	aaACACGtaactg	1614	-	ctttggtatCAAGTt
1666	+	aaacACGTAact	1664	-	caaaaCACGTaactg
1667	+	aacACGTAac	1667	-	aacACGTAac
1667	+	aACACGtaactg	1667	-	aacACGTAactg
1667	+	aacACGTAact	1667	-	aACACGtaactg
1667	+	aacACGTAactgt	1668	-	ACACGtaactg
1667	+	aACACGtaact	1669	-	cACGTAac
1668	+	acACGTAactg	1770	-	tataaCTTTTaacatttttat
1669	+	CACGT	1898	-	atggctaattagACACGaaat
1670	+	ACGTA	1910	-	ACACGaaataa
1902	+	ctaattagACACGaaa	1948	-	acTTGACct
1908	+	agACACGaaata	1964	-	cctTTTCCttagt
1909	+	gACACGaaataa	1964	-	ccttTTCCTtagt
1909	+	gACACGaaata	1965	-	ctttTCCTTagtc
27	-	attgtTACGTaat	1967	-	ttTCCTTagtc
28	-	ttgttACGTAatt	1975	-	gtcgTTTCCtatt
29	-	tgttaCGTAAtt			

Table S8 List of NAC transcription factor binding sites in *SmTGA*.

Gene	*SmTGA*	
Position	Strand	Sequence
32	+	tTGCTTgatttcaatgatcca
33	+	TGCTTgatttcaatg

255	+	acatAAGGAaata
257	+	ataaGGAAAtaag
300	+	tctcAGATAccagt
695	+	CATGTg
820	+	acaagGGAAActa
1334	+	CATGTg
1385	+	aataaaatatcAAAAGtgagg
1410	+	aggagGGTAAaa
1422	+	taTACGTaga
1425	+	ACGTA
1604	+	ttTTACGatata
1863	+	attgaaaaaaaAAAAGagaaa
1896	+	aaaatgACGTG
1897	+	aaatgACGTGggggc
1909	+	ggctaGCGTAg
1909	+	ggctaGCGTAgtt
1910	+	gctaGCGTAgtt
1937	+	gtcACGTCtgtc
1939	+	CACGT
1958	+	tctcTTTCCctcg
1995	+	taTTAAGcaaaa
1995	+	taTTAAGcaaa
31	-	ttTGCTTgatttcaatg
32	-	tTGCTTgatttcaatg
33	-	TGCTTgatttcaatg
277	-	cttctgggatgcCAAGTttca
321	-	cACATG
338	-	acataCTTTTtctttatttct
542	-	tgtgaCTTTTtatgagtttta

821	-	caagGGAAActaa
863	-	atTTACCcttgg
1041	-	gaggagAATCTtaa
1041	-	gaggaGAATCttaa
1042	-	aggagAATCTtaaa
1043	-	ggaGAATCtt
1121	-	attcaCTTTTatttttaaatt
1184	-	atTTACCttcaa
1420	-	aatatACGTAgac
1422	-	tatACGTAga
1424	-	TACGT
1604	-	ttTTACGatata
1897	-	aaatGACGTggg
1902	-	ACGTG
1910	-	gctaGCGTAgtt
1937	-	gtcACGTCtgtc
1939	-	CACGTctgtct
1959	-	ctcTTTCCctcgt

Table S9 List of NAC transcription factor binding sites in *SmPAD4*.

Gene	*SmPAD4*		Gene	*SmPAD4*	
Position	Strand	Sequence	Position	Strand	Sequence
229	+	ttggaacaCAAGTttg	75	-	TACGT
493	+	caGTCAAgt	76	-	ACGTG
553	+	tgaattCGTAAct	225	-	cattttggaacaCAAGTttgg
600	+	tgtaGCGTGc	599	-	atgtaGCGTGcag
601	+	gtaGCGTGc	601	-	gtaGCGTGca
602	+	taGCGTGcag	1142	-	cataaCTTTTtaattttaatt
667	+	CATGTg	1233	-	acgtACGCAa

1110	+	CATGTg	1233	-	acgtaCGCAAacc
1233	+	ACGTA	1233	-	acgTACGCaaac
1233	+	acgtACGCAaacc	1233	-	acgTACGCaaac
1233	+	acgTACGCaaac	1233	-	acgTACGCaaacc
1233	+	acgtACGCAaacc	1234	-	cgtACGCAaa
1234	+	cgtACGCAaa	1234	-	cgTACGCaaa
1338	+	aggaAAGGAaagg	1235	-	gtaCGCAAac
1344	+	ggaaaGGAAAcag	1235	-	gtaCGCAAacc
1344	+	ggaaAGGAAacag	1235	-	gTACGCaaacc
1345	+	gaaaGGAAAcaga	1345	-	gaaaGGAAAcaga
1651	+	aTTACTtta	1380	-	cACATG
1691	+	aTTACTttt	1436	-	cACATG
1794	+	aTTACTcat	1690	-	aattaCTTTTtactttttaaa
35	-	tttAGTAAt	1697	-	ttttaCTTTTtaaaaacttta

Table S10 List of plant morbidity and disease index for testing the resistance of E31 and E32 to *Ralstonia solanacearum*.

Material	Total	Disease plants	Uninfected plants	Incidence (%)	Evaluation of scale					Disease index
					0	1	2	3	4	
E31	81	9	72	11.11	72	9	0	0	0	2.78
E32	73	65	8	89.04	8	18	22	23	2	47.6

Table S11 List of primers used for RT-qPCR.

Primer	Sequence
qPCR-*SmDDA1b*-Forward	CCTCCGAACAATGCCACA
qPCR-*SmDDA1b*-Reverse	GAAATCCCCTTGCCGTCT
qPCR-*SmNAC* -Forward	TCGTGGTAACGCCAAGGTTG
qPCR-*SmNAC* -Reverse	TTGGTCCATGCCGTTTGTAT

SmActin-Forward	GTCGGAATGGGACAGAAGGATG
SmActin-Reverse	GTGCCTCAGTCAGGAGAACAGGGT
SmCyclophilin-Forward	GCGCCAAATTCAAGGACGAGAACT
SmCyclophilin-Reverse	ACAGCCTCGGCCTTCTTAATCACA
MM-qPCR-*SmDDA1b*-Forward	TGACCATAGTTCAAAAGACGGC
MM-qPCR-*SmDDA1b*-Reverse	TAACCGTGCAATTAGTTCATCCT
SlActin-Forward	TTGCCGCATGCCATTCT
SlActin-Reverse	TCGGTGAGGATATTCATCAGGTT
SlGAPDH-Forward	ACCACAAATTGCCTTGCTCCCTTG
SlGAPDH-Reverse	ATCAACGGTCTTCTGAGTGGCTGT
qPCR-*ICS1*/*SlICS1*-Forward	GCATGGGACAATGCTGCTGCCTCATGGA
qPCR-*ICS1*/*SlICS1*- Reverse	TCTGGTGCTACGAGCAAGTACCACCT
qPCR-*SmEDS1/SlEDS1*-Forward	GTTTCGCAGACAAGTTGAGCC
qPCR-*SmEDS1/SlEDS1*-Reverse	CTCTGTGTGAACCGATAACGC
qPCR-*SmGluA/SlGluA*-Forward	GCCGACTGGGTGAGATGGTAA
qPCR-*SmGluA/SlGluA*-Reverse	ACATTGTTGTGCCCGTGGAC
qPCR-*SmNPR1/SlNPR1*-Forward	CTTGGACTGGGTGTTGCTAATG
qPCR-*SmTGA/SlTGA*-Forward	GCAAGTGACCCTGAACTACGAAG
qPCR-*SmTGA/SlTGA*-Reverse	GGGTTTTCCACATCCCTGACAAG
qPCR-*SmSGT1/SlSGT1*-Forward	TTCTCGGTTTTGAGGAAGGG
qPCR-*SmSGT1/SlSGT1*-Reverse	GCAGATACCAAGTGATGTCTACCA
qPCR-*SmPAD4/SlPAD4*-Forward	ACATCGGCTGAAACCTCCTTATT
qPCR-*SmPAD4/SlPAD4*-Reverse	TTTGATAAGTGGTGGGGAAATGA

Table S12 List of primers.

Primer	Sequence	Restriction sites	Plasmid
pTRV2-*SmDDA1b*-Forward	ggaattcCCTCCGAACAATGCCACA	*Eco*R I	pTRV2
pTRV2-*SmDDA1b*-Reverse	tcccccgggGAAATCCCCTTGCCGTCT	*Sma* I	pTRV2
pTRV2-Universal-Forward	TGAGGGAAAAGTAGAGAACG		pTRV2
pTRV2-Universal-Reverse	CCTATGGTAAGACAATGAGT		pTRV2
pEAQ-EGFP-*SmDDA1b*-Forward	ctgcccaaattcgcgaccggtATGGAGGATACCTCATCATCCATT	*Age* I	pEAQ-EGFP
pEAQ-EGFP-*SmDDA1b*-Reverse	gcccttgctcaccataccggtTGTGTCCCCCCTTAACCGTG	*Age* I	pEAQ-EGFP
pEAQ-EGFP-Universal-Forward	AGAGTTTTCCCGTGGTTTTCGAACT		pEAQ-EGFP
pEAQ-EGFP-Universal-Reverse	GGACACGCTGAACTTGTGGCCGTTT		pEAQ-EGFP
BiFC-*SmDDA1b*-Forward	tggcgcgccactagtggatccATGGAGGATACCTCATCATCCATT	*Bam*H I	pUC-SPYNE/YCE
BiFC-*SmDDA1b*-Reverse	agcggtaccctcgaggtcgacTGTGTCCCCCCTTAACCGTG	*Sal* I	pUC-SPYNE/YCE
BiFC-*DDB1*-Forward	tggcgcgccactagtggatccATGTCTAGGGAATGCCTCAGCG	*Bam*H I	pUC-SPYNE/YCE
BiFC-*DDB1*-Reverse	agcggtaccctcgaggtcgacATGCAACCTTGTCAACTCTTCAAC	*Sal* I	pUC-SPYNE/YCE
BiFC-*CUL4*-Forward	tggcgcgccactagtggatccATGTCTCAACCCACCACCAAAC	*Bam*H I	pUC-SPYNE/YCE
BiFC-*CUL4*-Reverse	agcggtaccctcgaggtcgacGTTAAACTCATGTTCTGCCACCTG	*Sal* I	pUC-SPYNE/YCE
BiFC-*SmNAC*-Forward	tggcgcgccactagtggatccATGGGTGTTCAAGAAAAAGATCCT	*Bam*H I	pUC-SPYNE/YCE
BiFC-*SmNAC*-Reverse	agcggtaccctcgaggtcgacCTACTGTCTGAACCCGAGATTTAACG	*Sal* I	pUC-SPYNE/YCE
BiFC-Universal-Forward	CTCTAGAGTTAACCGGGCTC		pUC-SPYNE/YCE

BiFC-Universal-Reverse	CCCGGGAGCGGTACCCTC		pUC-SPYNE/YCE
AD-*SmDDA1b*-Forward	gccatggaggccagtgaattcATGGAGGATACCTCATCATCCATT	*Eco*R I	pGADT7
AD-*SmDDA1b*-Reverse	cagctcgagctcgatggatccTCATGTGTCCCCCCTTAACCG	*Bam*H I	pGADT7
AD-*CUL4*-Forward	gccatggaggccagtgaattcATGTCTCAACCCACCACCAAAC	*Eco*R I	pGADT7
AD-*CUL4*-Reverse	cagctcgagctcgatggatccTTAGTTAAACTCATGTTCTGCCACC	*Bam*H I	pGADT7
AD-*SmNAC*-Forward	gccatggaggccagtgaattcATGGGTGTTCAAGAAAAAGATCCT	*Eco*R I	pGADT7
AD-*SmNAC*-Reverse	cagctcgagctcgatggatccCTACTGTCTGAACCCGAGATTTAACG	*Bam*H I	pGADT7
BD-*DDB1*-Forward	atggccatggaggccgaattcATGTCTAGGGAATGCCTCAGCGACCAAA	*Eco*R I	pGBKT7
BD-*DDB1*-Reverse	ccgctgcaggtcgacggatccCTAATGCAACCTTGTCAACTCTTCAACT	*Bam*H I	pGBKT7
BD-$SmNAC_{1\text{-}139}$-Forward	atggccatggaggccgaattcATGGGTGTTCAAGAAAAAGATCCT	*Eco*R I	pGBKT7
BD-$SmNAC_{1\text{-}139}$-Reverse	ccgctgcaggtcgacggatccTAATCTATATTCATGCATGATCCAATTAG	*Bam*H I	pGBKT7
AD-Universal-Forward	ATGGCCATGGAGGCCAGTGAATTC		pGADT7
AD-Universal-Reverse	TGCAGCTCGAGCTCGATGGATCCC		pGADT7
BD-Universal-Forward	CGACTCACTATAGGGCGAGCCGCC		pGBKT7
BD-Universal-Reverse	GGAATTAGCTTGGCTGCAAGCGCGC		pGBKT7
pEAQ-*SmDDA1b*-Forward	ttctgcccaaattcgcgaccggtATGGAGGATACCTCATCATCC	*Age* I	pEAQ
pEAQ-*SmDDA1b*-Reverse	tgatggtgatggtgatgcccgggTGTGTCCCCCCTTAACC	*Sma* I	pEAQ
pEAQ-Firefly-*SmNAC*-Forward	ggaaagatcgccgtgctcgagATGGGTGTTCAAGAAAAAGATCCT	*Xho* I	pEAQ-Firefly
pEAQ-Firefly-*SmNAC*-Reverse	accagagttaaaggcctcgagCTACTGTCTGAACCCGAGATTTAACG	*Xho* I	pEAQ-Firefly

pEAQ-GFP-*SmNAC*-Forward	tattctgcccaaattcgcgaccggtATGGGTGTTCAAGAAAAAGATC	*Age* I	pEAQ-GFP
pEAQ-GFP-*SmNAC*-Reverse	aaagttcttctcctttgctagtcatCTGTCTGAACCCGAGAT	*Age* I	pEAQ-GFP
pEAQ- Universal-Forward	TCTTCTTCTTGCTGATTGGT		
pEAQ- Universal- Reverse	ACCTGCTAAACAGGAGCTC		
pEAQ-Firefly- Universal-Forward	GTCGCCAGTCAAGTAACAACCG		
pEAQ-Firefly- Universal-Reverse	ACCTGCTAAACAGGAGCTC		
pEAQ-GFP- Universal-Forward	TCTTCTTCTTGCTGATTGGT		
pEAQ-GFP- Universal-Reverse	ATTAACATCACCATCTAATTC		
pAbAi-*SmDDA1bpro-1*-Forward	aaatgatgaattgaaaagcttTTCTGTTTGGGAGGCTACTTTTAAC	*Hin*d III	pAbAi
pAbAi-*SmDDA1bpro-1*-Reverse	accgagctcgaattcaagcttGAAATAGTTAAGGTCTTTTGGACGAA	*Hin*d III	pAbAi
pAbAi-*SmDDA1bpro-2*-Forward	aaatgatgaattgaaaagcttCTATTTCTTGGTACAGTAATATATTATCT	*Hin*d III	pAbAi
pAbAi-*SmDDA1bpro-2*-Reverse	accgagctcgaattcaagcttTTTGAAAAAAAATTGGACCATAAAA	*Hin*d III	pAbAi
pAbAi-*SmDDA1bpro-3*-Forward	aaatgatgaattgaaaagcttTATCGCGCATAATAAAAACTTAAT	*Hin*d III	pAbAi
pAbAi-*SmDDA1bpro-3*-Reverse	accgagctcgaattcaagcttAATACTGTTTCGTGGGGGCG	*Hin*d III	pAbAi
pAbAi-Universal-Forward	TCTAAGTCTGTGCTCCTTCC		
pAbAi-Universal-Reverse	CTTGTTTCTAAATCGGCTAC		
0800-*SmDDA1bpro*-Forward	ctatagggcgaattgggtaccTTCTGTTTGGGAGGCTACTTTTAAC	*Kpn* I	pGreen II 0800-Luc
0800-*SmDDA1bpro*-Reverse	tgtttttggcgtcttccatggAGTTGCCGCACAGCAAAATT	*Nco* I	pGreen II 0800-Luc
62-SK-*SmDDA1b*-Forward	cgctctagaactagtggatccATGGAGGATACCTCATCATCCATT	*Bam*H I	pGreen II 62-SK

62-SK-*SmDDA1b*-Reverse	gataagcttgatatcgaattcTCATGTGTCCCCCCTTAACCG	*Eco*R I	pGreen II 62-SK
62-SK-*SmNAC*-Forward	cgctctagaactagtggatccATGGGTGTTCAAGAAAAAGATCCT	*Bam*H I	pGreen II 62-SK
62-SK-*SmNAC*-Reverse	gataagcttgatatcgaattcCTACTGTCTGAACCCGAGATTTAACG	*Eco*R I	pGreen II 62-SK
0800-Universal-Forward	GAAAGGGGGATGTGCTGCAAGGCG		pGreen II 0800-Luc
0800-Universal-Reverse	CTGCCAACCGAACGGACATT		pGreen II 0800-Luc
62-SK-Universal-Forward	ACGTTCCAACCACGTCTTCA		pGreen II 62-SK
62-SK-Universal-Reverse	TTATCGGGAAACTACTCACA		pGreen II 62-SK
R. solanacearum check -Forward	GTCGCCGTCAACTCACTTTCC		
R. solanacearum check -Reverse	GTCGCCGTCAGCAATGCGGAATCG		
